# A success targeted nano delivery to lung cancer cells with multi-walled carbon nanotubes conjugated to bromocriptine

**DOI:** 10.1038/s41598-021-03031-2

**Published:** 2021-12-24

**Authors:** Fatemeh Mohammadhosseini Kamazani, Fattah Sotoodehnejad nematalahi, Seyed Davar Siadat, Majid Pornour, Mojgan Sheikhpour

**Affiliations:** 1 Department of Biology, Science and Research Branch, Islamic Azad University, Tehran, Iran; 2 Department of Mycobacteriology and Pulmonary Research, Pasteur Institute of Iran, Tehran, Iran; 3 Microbiology Research Center, Pasteur Institute of Iran, Tehran, Iran; 4 Division of Endocrinology, Diabetes, and Nutrition, Department of Medicine, University of Maryland, College Park, USA

**Keywords:** Biotechnology, Cancer therapy, Drug delivery

## Abstract

In this research, a new nano drug-based multi-walled carbon nanotubes (MWCNTs) was prepared and evaluated qualitatively. Bromocriptine (BRC) was conjugated to functionalized carbon nanotubes. Then, the CHNS, FT-IR, SEM, and RAMAN tests for characterization of the conjugated drug were done. The nanofluid-containing nano-drug was evaluated on lung cancer cells (A549 & QU-DB) and MRC5 by MTT and flow cytometry tests. Then, the gene expression studies of dopamine receptor genes were done before and after nano-drug treatment. After that, a western blotting test was carried out for further investigation of dopamine receptors protein production. Finally, Bax and Bcl-2 secretion were measured by the ELISA method in cells affected by MWCNTs-BRC Nf compared to untreated cells. The results showed that the nano-drug had a significant lethal effect on cancer cells, while it had no toxicity on MRC5. Also, the nano-drug could significantly induce apoptosis in lung cancer cells at a lower dose compared to the drug alone. In this study, a targeted nano-drug delivery system was designed, and its performance was evaluated based on neurotransmitter pathways, and the results showed that it may be useful in the treatment of lung cancer. However, additional studies on animal models are underway.

## Introduction

Lung cancer is one of the most prevalent cancers and the leading cause of death worldwide. Recently, the relationship between the nervous and immune systems for identifying risk factors for lung cancer has been considered. In the past, neurotransmitters were known exclusively as transmitters of messages between cells within the nervous system. Still, today it is believed that these mediators are involved in regulating the immune and the nervous systems. However, the mechanism of this regulation is not fully understood. New evidence sheds more light on the role of neurotransmitters in immune function and regulating the migration of lymphocytes and tumor cells. Evidence of growing researches toughly supports the notion that dopamine receptors (DRDs) are associated with the regulation of tumor activities, such as tumor apoptosis, proliferation, invasion, and migration, which can limit tumor progress through triggering tumor immunity^1^.

DRD2 subtype is the main target for most anti-neurological drugs^2^. The role of DRD2 signaling in several malignant tumors has been investigated; however, the results are contradictory. Increased levels of DRD2 expression are associated with the development of several tumors, and some DRD2 antagonists have been used to reduce the progress of these malignancies^3–5^. Conversely, it has been revealed that DRD2 agonist has inhibitory properties in several cancer types, such as lung cancer, gastric cancer, prostate cancer, and leukemia^6–8^.

DRDs have five subgroups, including D1, D2, D3, D4, and D5, which have inhibitory or stimulatory effects depending on the type of receptors. Bromocriptine (BRC) is a dopamine-specific agonist and was accepted as the first therapeutic agent for adenomas. BRC binds to receptor D2 and inhibits prolactin secretion, and is used to treat Parkinson’s disease, type 2 diabetes, hyperprolactinemia, cocaine addiction, and neuroleptic malignant syndrome^9^.

These conditions are due to a decrease in dopamine secretion from a specific area of the brain. BRC is an accepted therapeutic agent to reduce the secretion of the growth hormone prolactin and reduce cell proliferation and tumor cell size^10^. Previous studies have shown that the expression profile of dopamine receptor genes in people with lung cancer is different from that in healthy people. This profile is changed after the patient recovers from the disease^11^. BRC induced significant cell apoptosis in a cell line of lung adenocarcinoma. It inhibited the proliferation of lung cancer cells in a dose-dependent manner. At the effective dose of the drug, the expression of D2 receptor genes increased appropriately after treatment^12^. In another study, BRC-based liposomal nanosystems were designed and fabricated to act purposefully through a specific receptor and increase drug penetration. The nano liposome-containing BRC caused about 50% of apoptosis in lung cancer cells^13^. Common treatments of lung cancer include drug treatment, radiotherapy, chemotherapy, surgery and targeted therapy, and immunotherapy. Recently, new methods, such as drug delivery systems and the use of nano drugs, have been widely considered.

Various nanomaterials, such as carbon nanotubes (CNTs), have been used to treat and diagnose cancers^14^. CNTs are allotropes of carbon that, in addition to their ability as carriers for a wide range of therapeutic molecules, their high surface area and the ability to manipulate physical surfaces and dimensions make them suitable for thermal conductivity to kill cancer cells. CNTs are divided into two main categories: single-walled carbon nanotubes (SWCNTs) and multi-walled carbon nanotubes (MWCNTs), and can play a key role in biomimetic nanomedicine toward personalized medicine for different diseases^15,16^.

Many studies have been conducted on different biomedical effects of MWCNTs in several diseases and cell signaling pathways such as apoptosis and autophagy. The results of extensive investigations show that MWCNTs have high efficient drug loading capacity. They could be used as proper drug nanocarriers and promising nanoplatforms for cancer therapy. Besides, to improve their biocompatibility and conjugation capacity, and reduce their toxicity, they are functionalized with chemical groups such as hydroxyl or carboxyl^17,18^.

This study aimed to prepare a nano-drug with a high and targeted anti-cancer effect and also to deliver it to cancer cells with higher efficiency compared to the free drug. Also in this research by functionalization of MWCNTs, conjugation of drug, and preparation of nanofluid containing this nano-drug, it was tried to increase the stability of the drug in nanofluid and reduce its toxicity on normal cells compared to cancer cells.

## Materials and methods

### Preparation of nanotubes and drug, and functionalization of MWCNTs

Functionalized multi walled carbon nanotubes (MWCNTs-COOH) and BRC were prepared from the US Research Nanomaterials, USA and Iran Hormone Company, respectively. For acylation of carboxylated MWCNTs, 1 g of carboxylated MWCNTs with 20 ml of thionyl chloride (Merk, Germany) was mixed at 60 °C for 14 h in the reflux system. The final product was washed and separated with tetrahydrofuran (Merk, Germany) and acetone, and then, it was dried in an oven at 70 °C.

### Drug conjugation, nano-drug characterization, and nanofluid preparation

In this step, 3 g of the drug and 1 g of chlorate MWCNT powder were added to 50 ml of dimethylformamide (DMF) solution. The solution was refluxed at 60 and 70 °C for 36 h and was extracted with tetrahydrofuran, methanol (96%), and ethanol, and then the precipitate was dried at room temperature. Elemental analysis of C-H-N-S, FT-IR, and RAMAN tests were conducted to ensure the binding of the drug and CNTs. Observation of the functionalized nanotubes with drug and nanotubes was done using a scanning electron microscope (SEM). For nanofluid preparation, 0.2 g of nano-drug powder, 6 ml of 96% ethanol, and 0.06 g of Arabic gum were added to 100 ml of deionized water. The mixture was stirred for 20 min and was placed in an ice container, and then, sonication was carried out for 20 min at 200 W^19^.

### Cell culture

Two human lung cancer cell lines QU-DB (human large cell carcinoma line), A549 (human adenocarcinoma lung cancer cell lines), and MRC5 (human non-malignant lung fibroblast) as a control normal cell line were applied from the National Cell Bank, Pasteur Institute, Iran. A549 and QU-DB cells were cultured in DMEM (Dulbecco's Modified Eagle Medium) (Biosera, cat no: LMD1111, France), and MRC5 cell was cultured in RPMI1640 (Biosera, cat no: LMR1638, France) complemented with 10% Fetal Bovine serum, penicillin, and streptomycin antibiotics (Biosera, UK) and incubated at 37 °C with 5% CO2.

### In vitro cytotoxicity and apoptosis assays

To investigate the effect of conjugated nano-drug formulation on enhancing the cytotoxicity outcomes, in vitro cytotoxicity profile of free BRC, functionalized MWCNTs Nf, and MWCNTs-BRC Nf using MTT assay in triplicate was conducted on A549, QU-DB, and MRC5 cell lines^20^. About 10^4^ cells were seeded in each well of 96-well plates in DMEM containing 10% FBS, and the cell was allowed to adhere and achieve the suitable confluence overnight with 5% CO2 at 37 °C in a humid CO2 incubator. Next, the medium was removed, and cells were exposed to drugs for 24–48 h, regarding the culture condition with different equal doses for MWCNTs Nf, MWCNTs-BRC Nf (0, 20,40, 80, 100, 200, 400, 800, 1200, 1600, 2000 μg/ml), and free BRC (0,18, 36, 72, 144, 288, μmol/l). Then, half-maximal inhibitory concentration (IC_50_) values were estimated in different groups within 24 and 48 h with 95% confidence intervals (95% CI). To investigate the level of apoptosis and necrosis induced by active targeted MWCNTs-BRC Nf, according to IC_50_ values in different groups of cell lines, treatments with equal appropriate concentration (50 μg/ml) was performed. In addition, regarding a previous study, treatments of BRC (72 μmol/l) and combination treatment of equivalent amounts of BRC (72 μmol/l) + F-MWCNTs Nf (120 μg/ml) in the fresh medium was performed. After 48 h, the cells were detached and washed with PBS, and then, suspended in 500 ml of 1X Binding buffer. Then, 10 μl of FITC-Annexin V was added to the cells, and subsequently, 10 μl propidium iodide (PI) solution was added. In the next step, the sample tubes were incubated for 10 min at 4 °C in the dark, and subsequently, the sample tubes were evaluated by a flow cytometer (FACS Calibur, BD biosciences, San Jose, CA, USA).

### Genes and proteins expression studies

To evaluate the changes in the expression of DRD1-DRD5 genes before and after treatments, the total RNA was extracted from all treated cells (GeneAll^®^ Hybrid-RTM kit), and cDNA synthesis and real-time PCR analysis were performed for DRD1-DRD5 and β-Actin, as an internal control gene. Table 1 presents the used specific primers. According to IC_50_ values with 95% CI in different groups, ELISA analysis was performed to analyze Bax and Bcl2 protein expression and the absorption of the standards and samples at a wavelength of 470 nm. Also, for each group, western blot analysis was performed for the evaluation of DRD1-DRD5 protein expression^21,22^.

**Table 1:** The primer sequences of DRD1-DRD5 and β-actin.

Locus	Primer (forward)	Primer (Reverse)	Product (bp)
β-Actin	5′-AGACGCAGGATGGCATGGG-3′	5′-GAGACCTTCAACACCCCAGCC 3′	161
DRD1	5′-CTTCCTCAACGTTTCGGAGCC-3′	5′-AGCTCTCCAAACGCCTTGCCTT-3′	100
DRD2	5′-TGTACAATACGCGCTACAGCTCCA-3′	5′-ATGCACTCGTTCTGGTCTGCGTTA-3′	127
DRD3	5′-TCTGTGCCATCAGCATAGACAGGT-3′	5′-TAAAGCCAAACAGAAGAGGGCAGG-3′	156
DRD4	5′-TCTTCGTCTACTCCGAGGTCCA-3′	5′-TGATGGCGCACAGGTTGAAGAT-3′	125
DRD5	5′-TCATCTATGCCTTCAACGCCGACT-3′	5′-AGCTGCGATTTCCTTGTGGAAGAC-3′	120

### Transmission electron microscopy (TEM) study

After culturing A549 cancer cells and reaching the appropriate surface confluence, a cell culture flask was treated according to the IC_50_ value of nano-drug (MWCNTs-BRC Nf) for each ml of cell culture (50 μg/ml), and after 48 h, it was examined by TEM. Cell suspension of A549 cell line containing at least 2.5 × 10^6^ cells was centrifuged for 15 min at 3000 rpm, and then, the primary fixation was performed, as previously described^23^.

### Statistical analysis

Statistical examinations were performed using Graph Pad Prism version 9.0.0. From triplicate conducted experiments, dose–response curves and IC_50_ values with 95% confidence intervals (95% CI) in each group and cell line were estimated. One-way ANOVA and Tukey’s post hoc test were used to compare the results of the groups. A *P* value less than 0.05 was considered significant. Furthermore, the rate of cell apoptosis and necrosis was analyzed by Flowojo software version 7.6. All results are expressed as mean ± standard deviation.

## Results

### Nano-drug characterization

**Figure 1:**
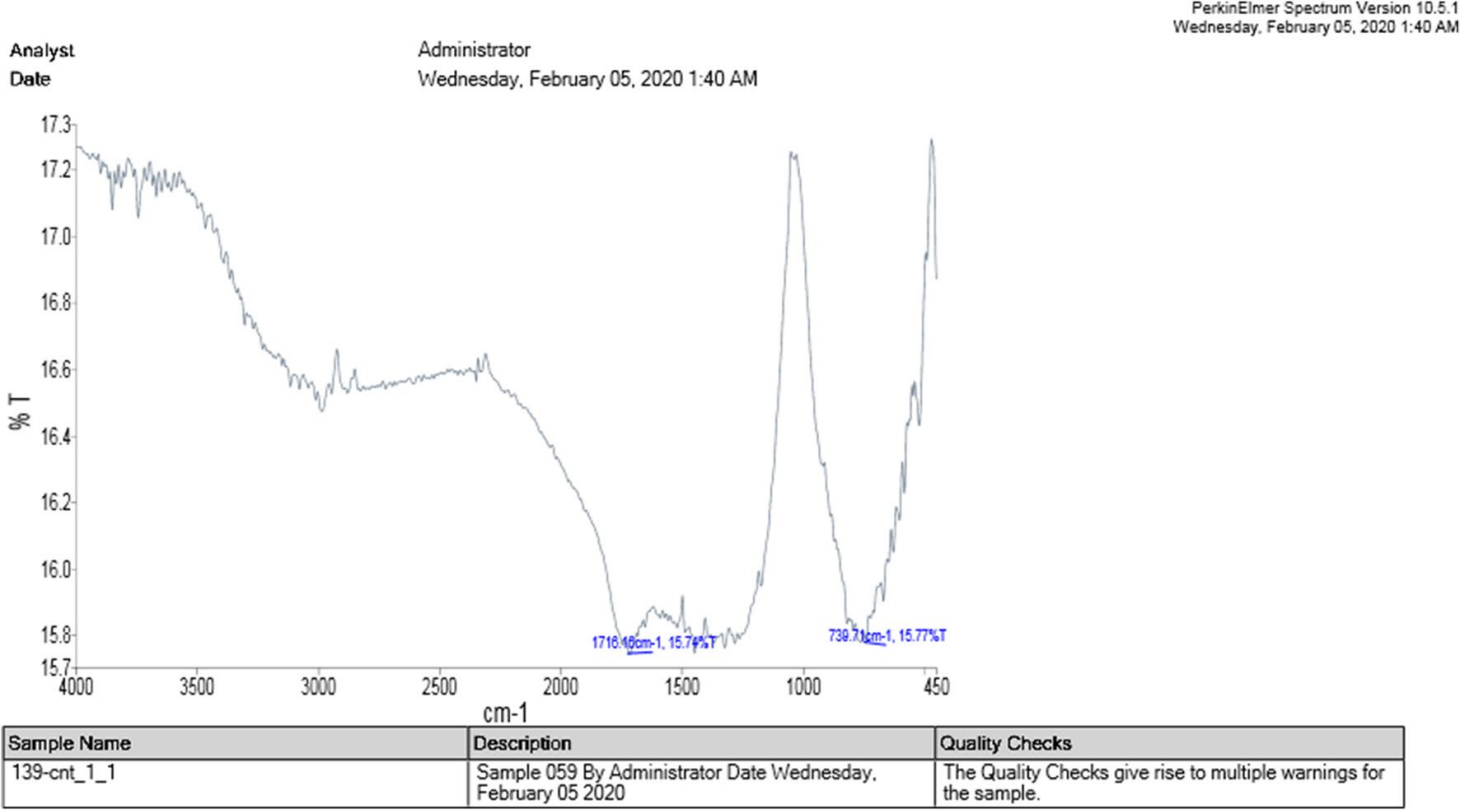
The infrared spectrum of carboxylated nanotubes.

**Figure 2:**
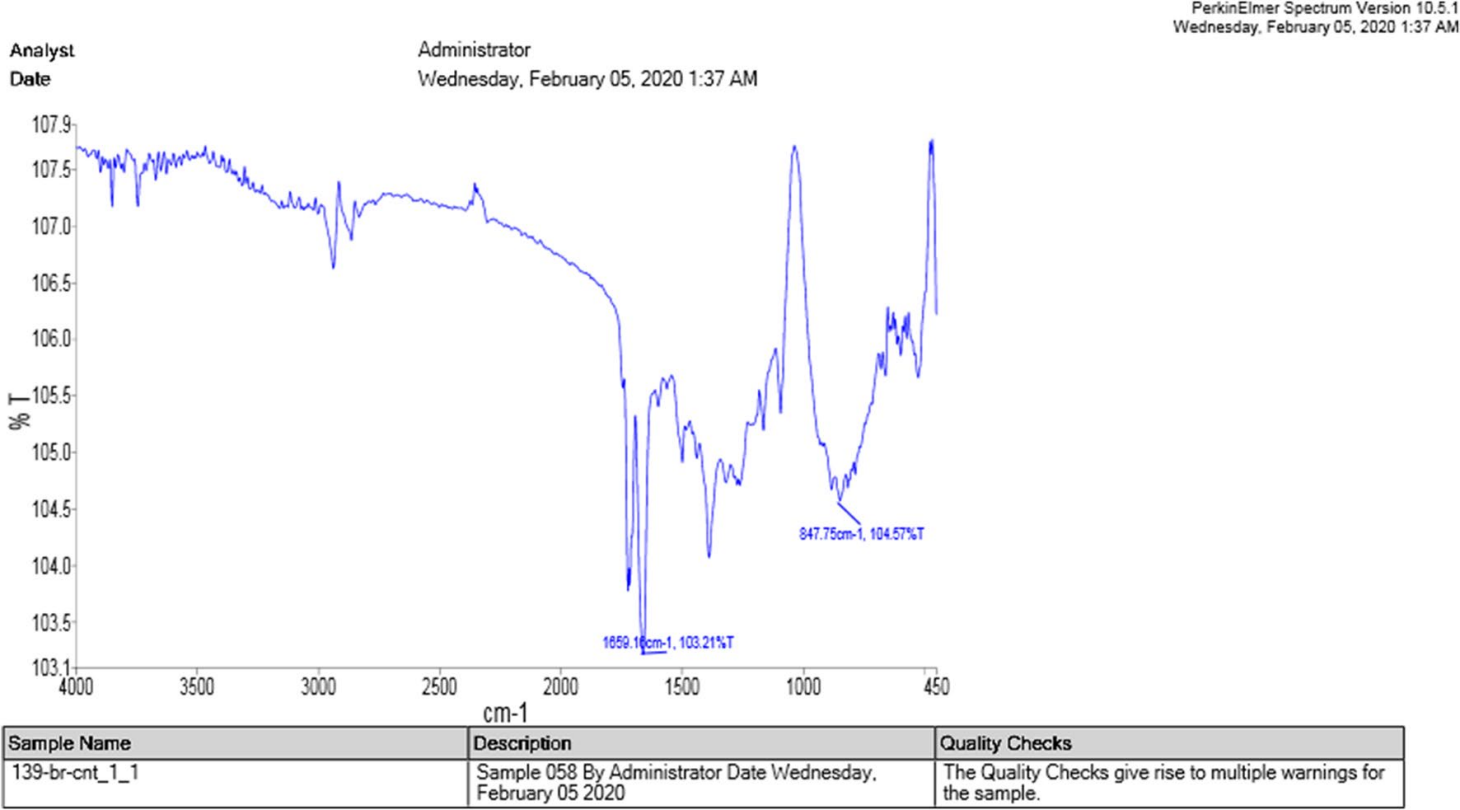
The infrared spectrum of nanotubes functionalized with BRC.

**Figure 3:**
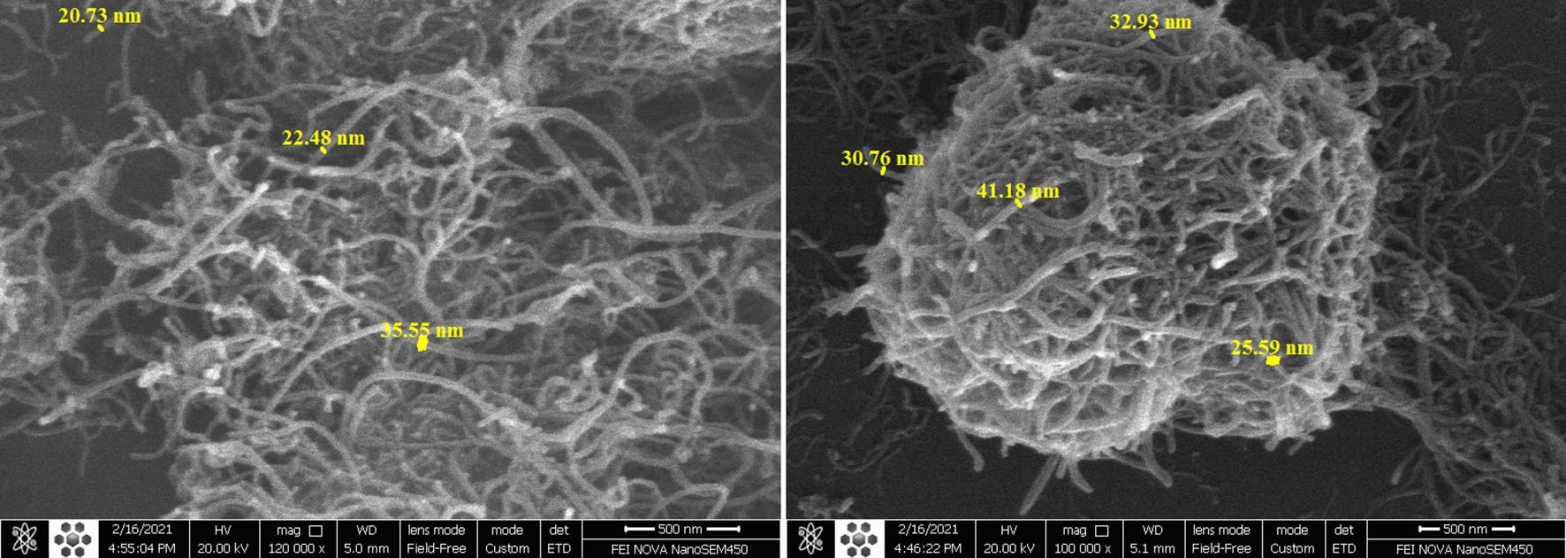
SEM images of the functionalized MWCNTs (left) and conjugated MWCNTs—BRC (Right). Original, unprocessed images are presented in Supplementary Figures S1–S4.

**Figure 4:**
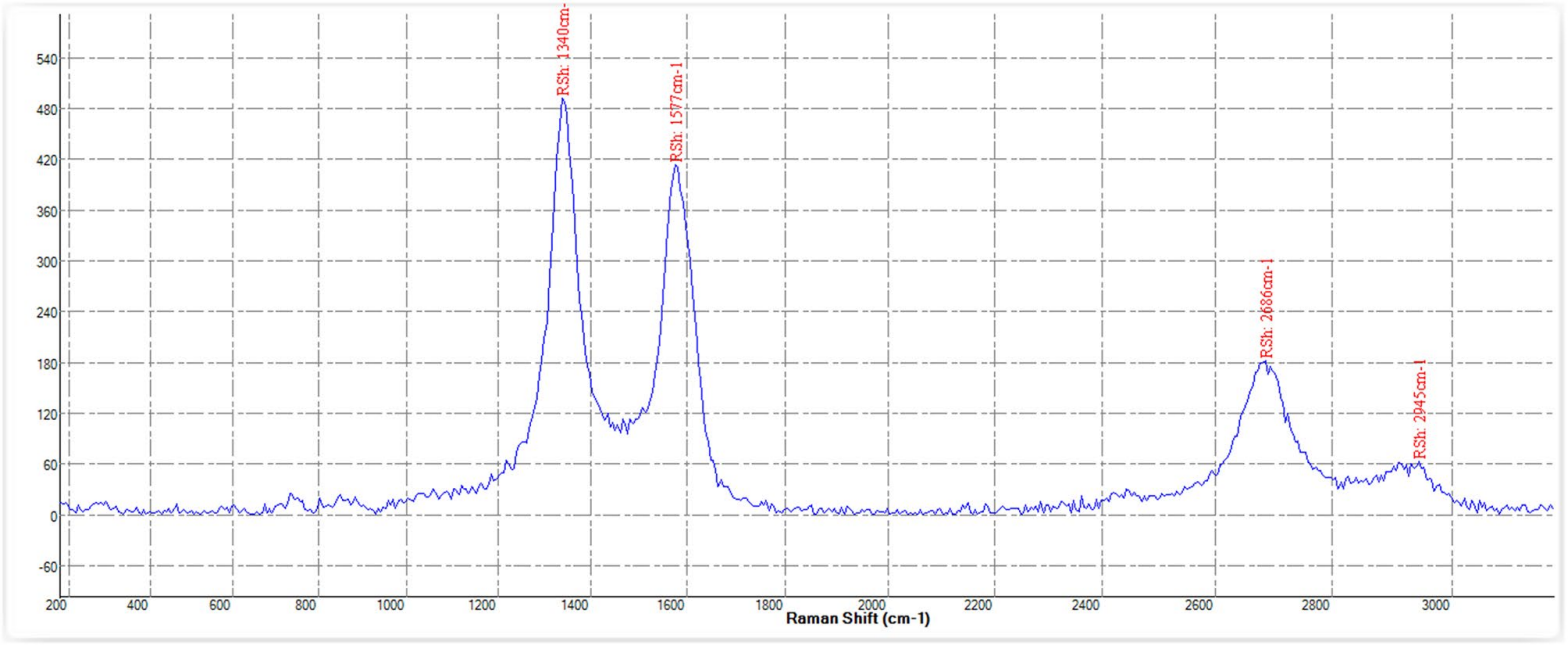
RAMAN structural information of the MWCNTs—BRC.

Table 2 and Figs. 1, 2, 3, 4 show the results of C-H-N-S, FT-IR, and RAMAN tests, and also SEM observation related to the conjugation of the drug. The results of the elemental analysis confirmed amid binding of drugs to the CNTs because detectable amounts of nitrogen compared to carboxylated CNTs did not contain any nitrogen. Considering the mass balance between these N contents and conjugated CNT, the loading of 0.2 mmol BRC/g of MWCNTs-BRC was found.

**Table 2:** The results of the elemental analysis of F-MWCNTs Nf, MWCNTs-BRC Nf and free BRC.

Sample name	%C	%H	%N	%S
F-MWCNTs Nf	88.37	0.32	–	–
MWCNTs-BRC Nf	45.09	0.78	1.03	–
Free BRC	51.61	5.94	8.52	4.20

The results of fourier-transform infrared spectroscopy (FTIR) spectrums of carboxylated and drug-functionalized nanotubes (Figs. 1, 2) indicated that the peak at 1659 cm^−1^ corresponded to an amide bond, which indicates the binding of the nano-tube to BRC.

The modifications of MWCNT samples were investigated by SEM. According to Fig. 3, (S1–S4) the drug-functionalized nanotubes had a different morphology and appear rougher compared to the naked nanotubes, which confirms that MWCNTs were functionalized with drugs. According to samples, a significant change in diameter was observed. The average diameter of the functionalized MWCNTs (chlorated) was 26.3 nm, whereas it improved in MWCNTs–BRC Nf to about 32.6 nm.

As shown in Fig. 4 (Raman spectroscopy of MWCNT–BRC), the D and G bands were at around 1330 cm^−1^ and 1590 cm^−1^, and the increase in the intensity of the defect mode bands at 1330 cm was related to the sp3 hybridization of carbon, which is used as evidence of the disruption of the aromatic system of π electrons by the attached molecules.

### In vitro cytotoxicity assay: MTT results regarding lung cancer and normal cell lines

**Figure 5:**
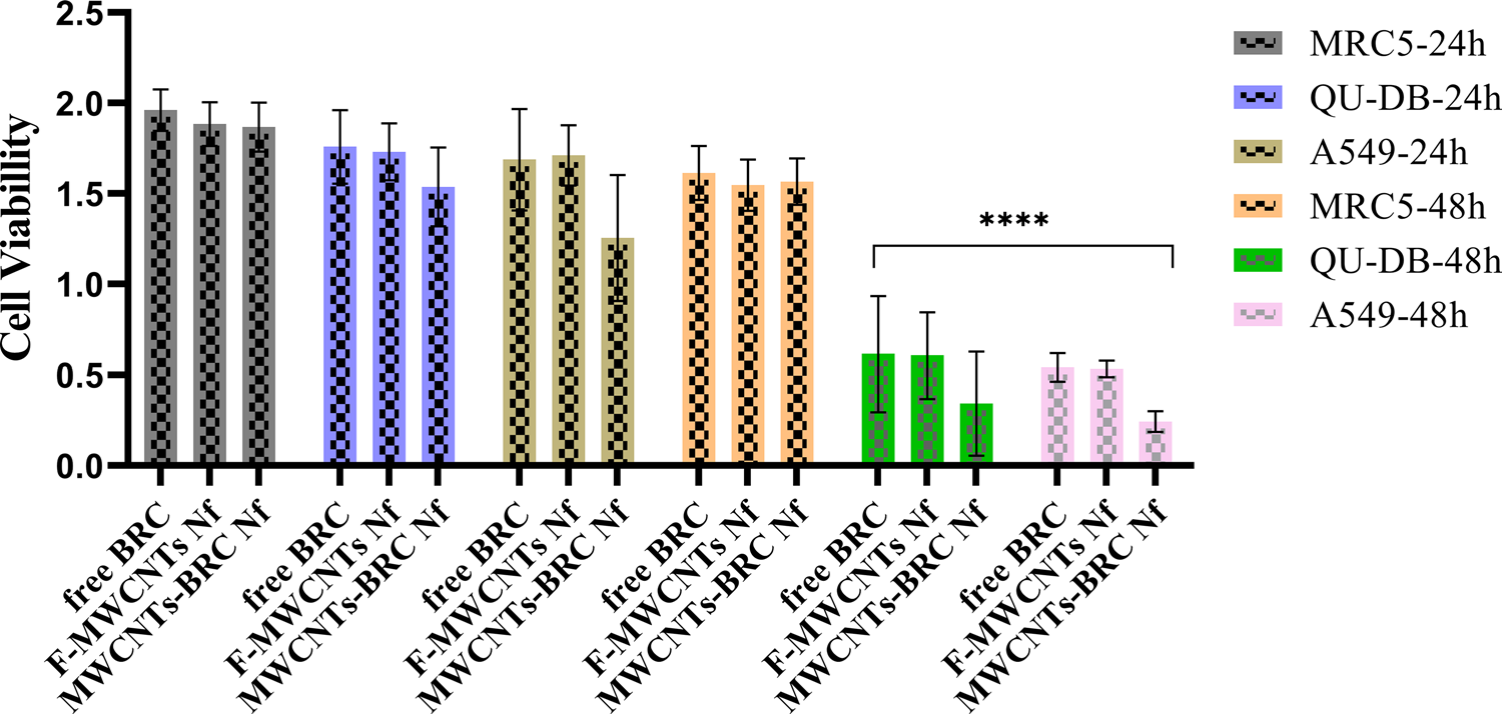
Comparison of antitumor activity in MRC5, A549, and QU-DB cell lines, treated with different concentrations of free BRC,F-MWCNTs Nf, and bromocriptine-conjugated MWCNTs nanofluid (MWCNTs-BRC Nf), in three different groups during 24 and 48 h.

As shown in Fig. 5, the results of MTT assay within 48 h incubation time after treatments, indicated that all groups, including free BRC, F-MWCNTs Nf, and MWCNTs-BRC Nf, significantly induced higher antitumor activity in both human lung cancer cell lines A549 and QU-DB compared to the normal control cell line MRC5. The results showed that MWCNTs-BRC Nf had a notable cytotoxic effect on cancer cell lines A549 and QU-DB within 48 h compared to 24 h significantly.

**Figure 6:**
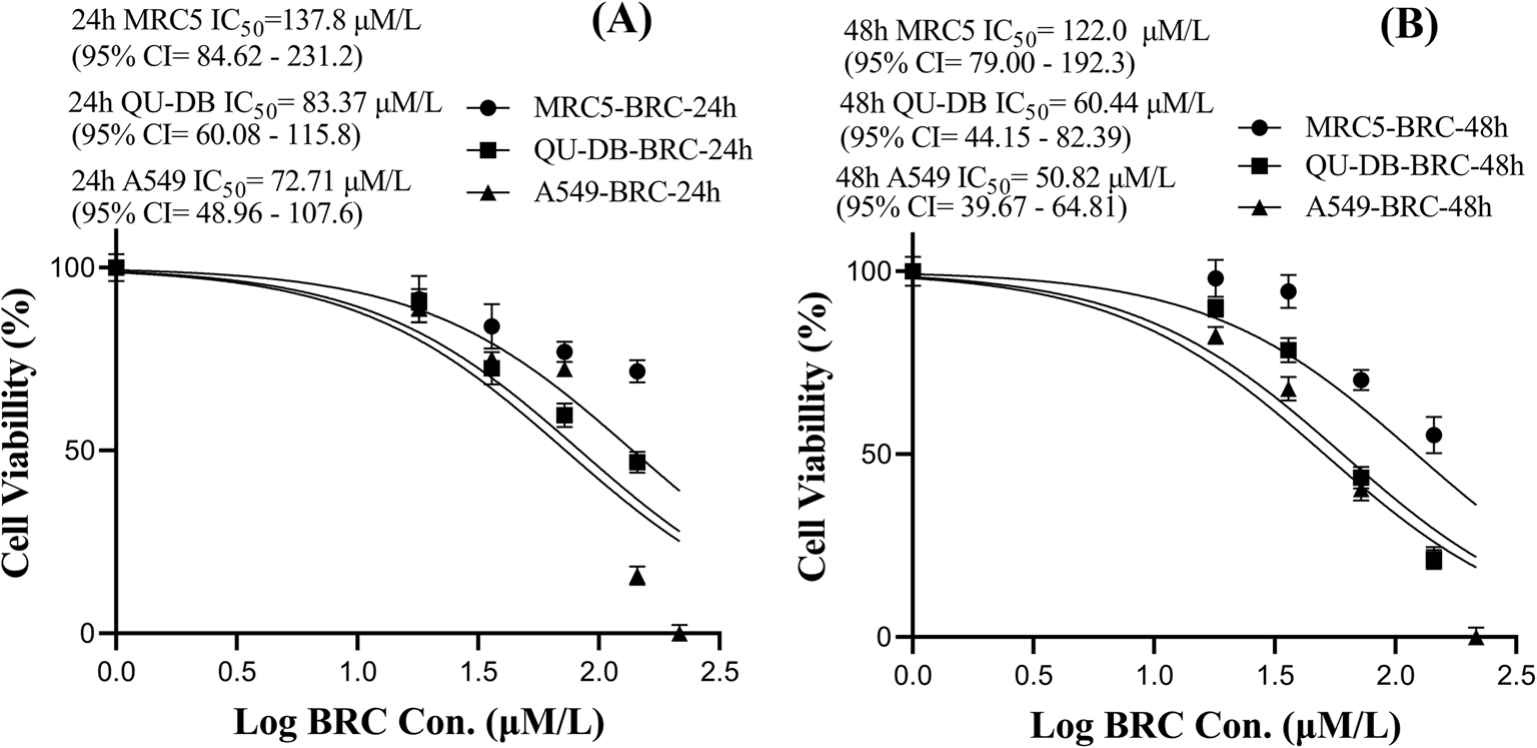
Comparison of the half maximal inhibitory concentration (IC_50_ values) in free BRC treated MRC5, QU-DB and A549 cell lines at 24 h (**A**) and 48 h (**B**).

**Figure 7:**
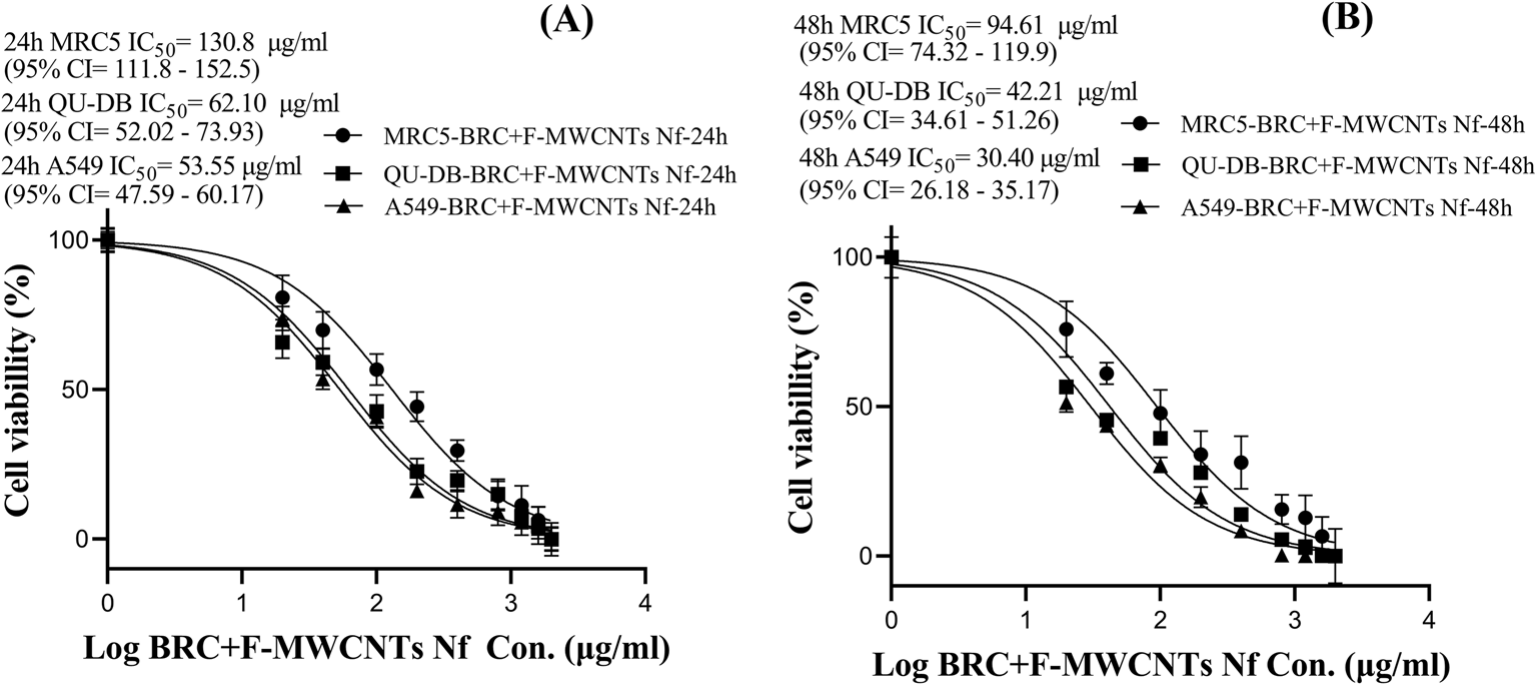
Comparison of the half maximal inhibitory concentration (IC_50_ values) in functionalized MWCNTs nanofluid (F-MWCNTs Nf) + BRC treated MRC5, QU-DB and A549 cell lines during 24 h (**A**) and 48 h (**B**).

**Figure 8:**
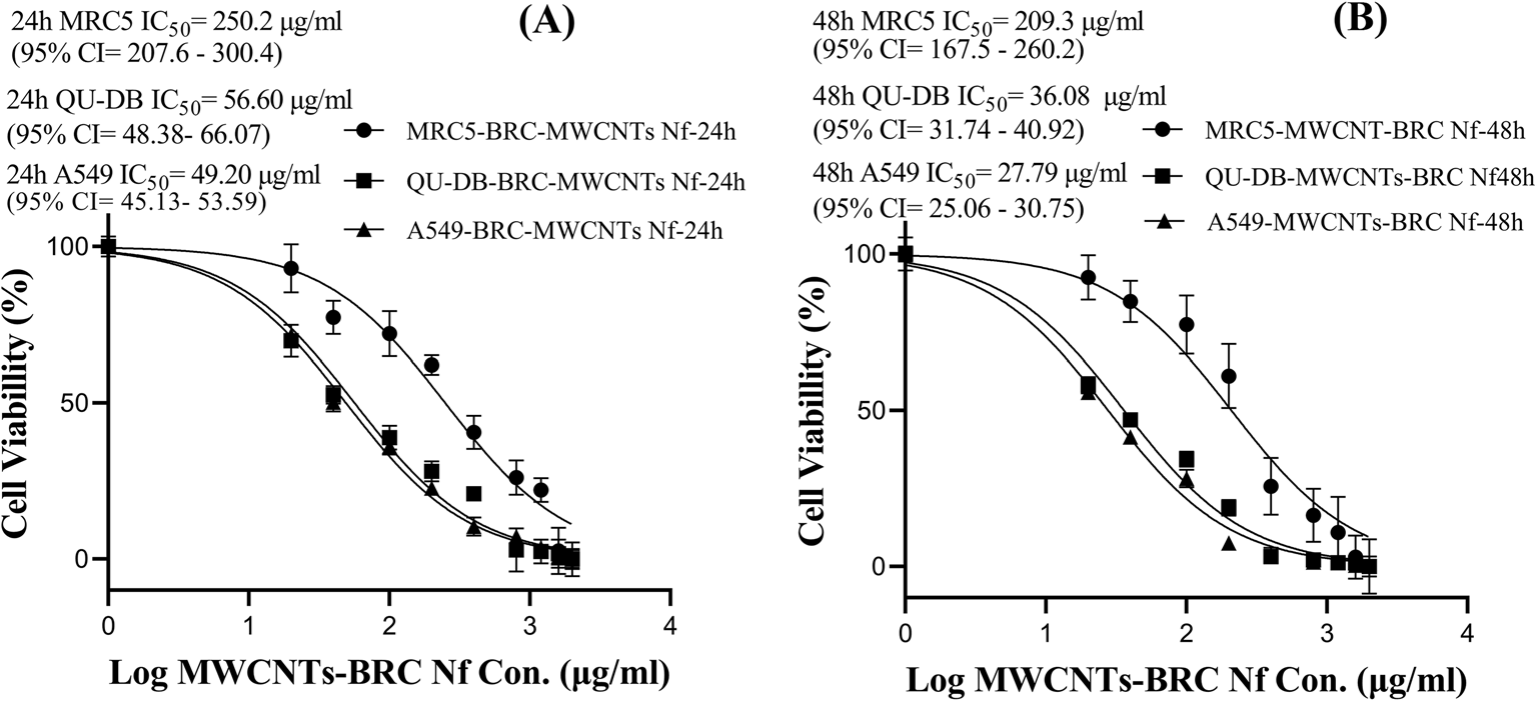
Comparison of the half-maximal inhibitory concentration (IC_50_ values) with 95% confidence intervals (95% CI) of bromocriptine-conjugated MWCNTs nanofluid(MWCNTs-BRC Nf) treated MRC5, QU-DB, and A549 cell lines during 24 h (**A**) and 48 h (**B**).

The one-way ANOVA results showed significantly lower cell viability in A549 and QU-DB cancer cell lines compared to the MRC5 cell line, especially in MWCNTs-BRC Nf-treated cell compared to F-MWCNT NFs- and free BRC-treated cells. The IC_50_ values (μg/ml) with 95% CI of all three forms of the drug were estimated after 24, and 48 h of incubation using MTT assay, and the results are shown using a nonlinear regression curve in Figs. 6, 7 and 8.

According to a previous study, in BRC-treated MRC5, QU-DB, and A549 cell lines during 24 h, the IC_50_ values were calculated to be 137.7 (95% CI 84.62–231.2), 83.37 (95% CI 60.08–115.8), and 72.71 (95% CI 48.96–107.6) μM/l, respectively. However, these values decreased during 48 h to 122.0 (95% CI 79.00–192.3), 60.44 (95% CI 44.15–82.39), and 50.82 (95% CI 39.67–64.81) μM/l, respectively.

IC_50_ values of the combination treatment with F-MWCNTs Nf and BRC in all three cell lines (MRC5, QU-DB, and A549) during 24 h were calculated to be 130.8 (95% CI 111.8–152.5), 62.10 (95% CI 52.02–73.93), and 53.55 (95% CI 47.59–60.17) μg/ml, respectively. These values decreased during 48 h to 94.61 (95% CI 74.32–119.9), 42.21 (95% CI 34.61–51.26), and 30.40 (95% CI 26.18–35.17) μg/ml, respectively. The IC_50_ of conjugated MWCNTs-BRC Nf during 24 and 48 h treatment decreased in both cancer cell lines, while the MRC5 normal cell line showed a lower reduction in IC_50_. The IC_50_ values of MWCNTs-BRC Nf-treated QU-DB and A549 cell lines were close to each other. The IC_50_ values of MWCNTs-BRC Nf-treated MRC5, QU-DB, and A549 cell lines within 24 h were calculated to be 250.2 μg/ml (95% CI 207.6–300.4), 56.60 (95% CI 48.38–66.07) and 49.20 (95% CI 45.13–53.59) μg/ml, respectively. These values decreased within 48 h to 209.3 (95% CI 167.5–260.2), 36.08 (95% CI 31.74–40.92) and 27.79 (95% CI 25.06–30.75) μg/ml, respectively.

### Apoptosis assay

**Figure 9:**
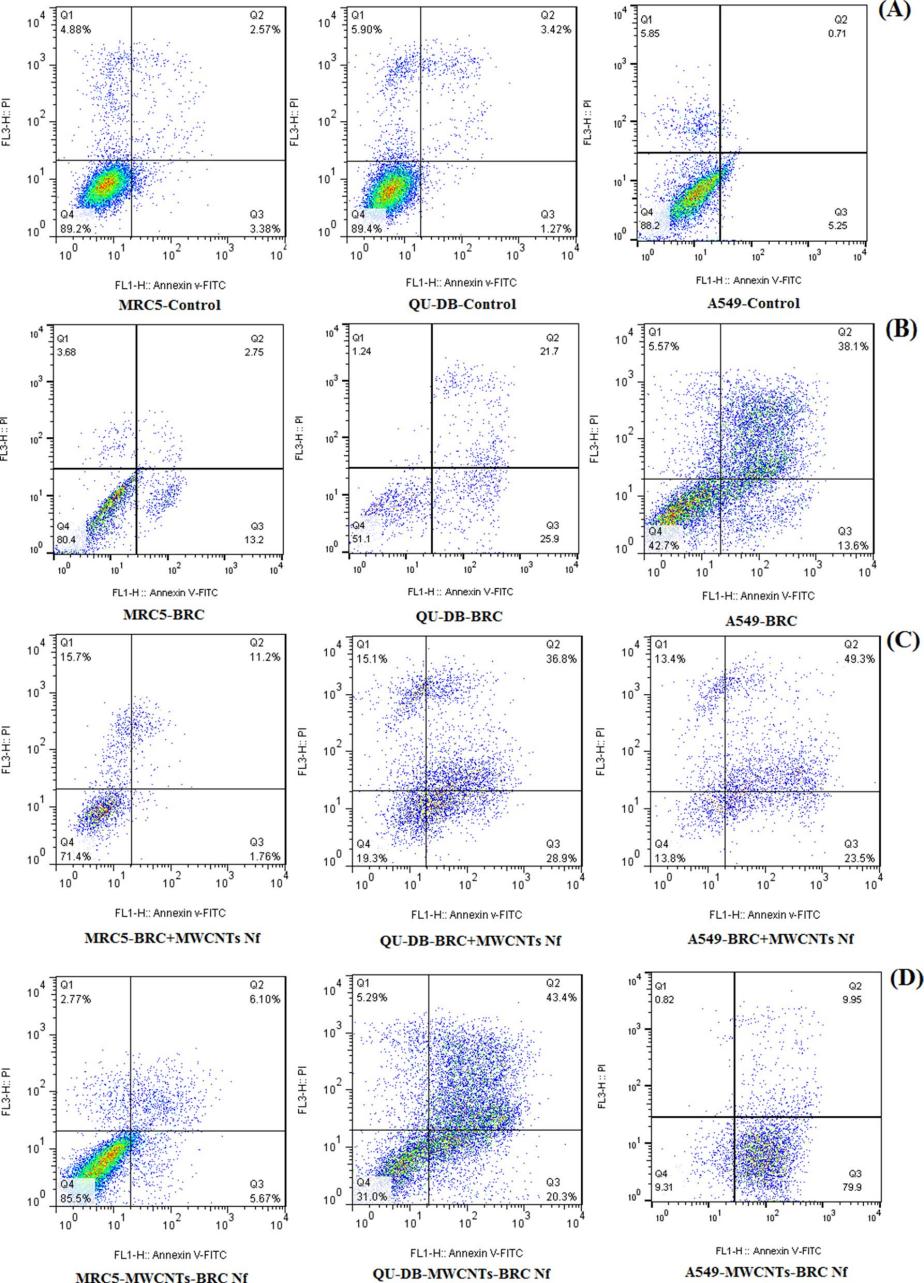
Flow cytometric analysis of apoptotic cells after treatments, 48 h’ incubation, and Annexin V/PI staining: the results related to MRC5, QU-DB, and A549 cells were shown from left to right as follow: control cells (**A**), Cells which were treated with free BRC (**B**), Cells which were treated with the combination of (BRC + MWCNTs) Nf (**C**), and cells which were treated with BRC conjugated MWCNTs nanofluid (MWCNTs-BRC Nf) (**D**).

Flow cytometry results showed that the conjugated MWCNTs-BRC Nf induced significantly greater total 90.67%, cell death in A549 (89.85% cell apoptosis) and 69% cell death in QU-DB (63.7% cell apoptosis) cancer cell lines compared to the 14.5% cell death in MRC5 (11.77% cell apoptosis) normal cell line (P < 0.0001). Moreover, cell necrosis in all three cell lines (MRC5 PI +  = 2.77%, A549 PI +  = 0.88%, and QU-DB PI +  = 5.29%) (Fig. 9) decreased compared to a previous study, in which cells were treated with a combination of F-MWCNTS Nf and BRC (MRC5 PI +  = 15.7%, A549 PI +  = 13.4%, and QU-DB PI +  = 15.1%). Both drug formulations (conjugated MWCNTs-BRC Nf and free BRC) significantly induced total cell apoptosis rates in both cancer cell lines compared to the MRC5 normal cell line. Flow cytometry results also showed that the total cell death in the A549 cell line was significantly higher than the QU-DB cell line.

### Gene expression, western blot, and ELISA analysis

**Figure 10:**
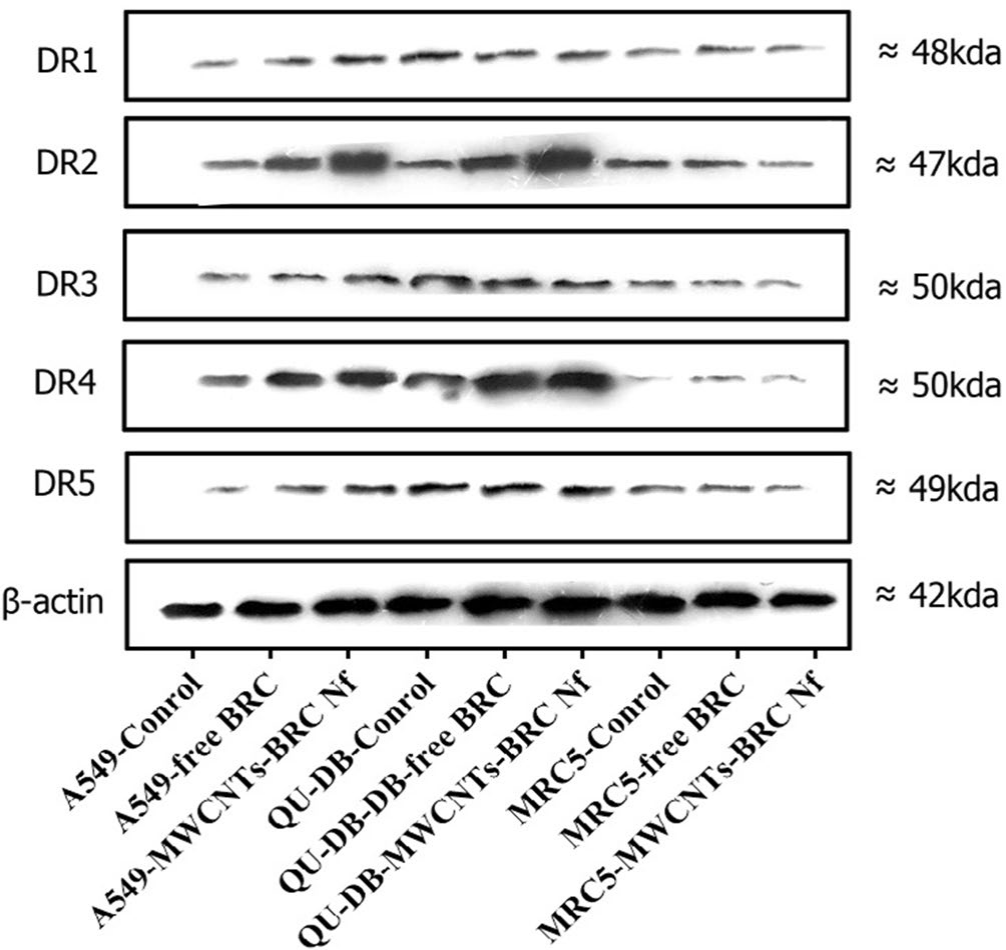
Effects of free BRC and active targeted nano-drug treatment (MWCNTs-BRC Nf) on the expression of DRD1-D5 on lung cancer cell lines (A549, QU-DB) and lung normal cell line (MRC5), respectively. From left to right for each cell line: control or untreated cells, treated cells with BRC and treated cells with MWCNTs-BRC Nf. The cell lysates prepared from cultured A549 QU-DB and MRC5 cell lines after 48 h treatment were subjected to Western blotting analysis. Results are representative of 3 independent experiments. Full-length blots/gels are presented in Supplementary Figures S5–S10.

**Figure 11:**
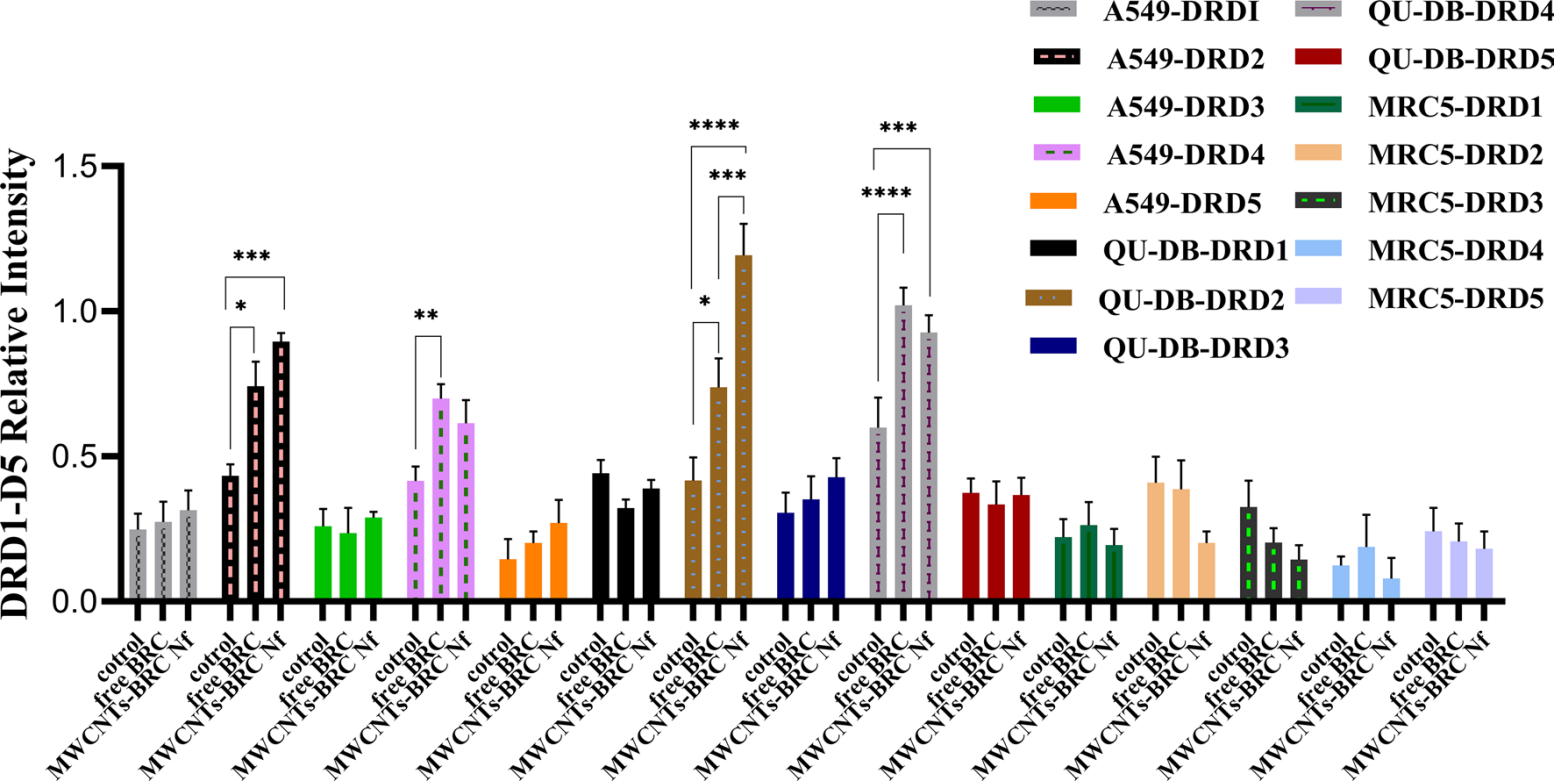
Overall comparison of expression profiles of different dopamine receptors at the protein level in the treated (free BRC and nano-drug) and untreated (control) forms in different normal and (MRC5) and lung cancer cells (A549 and QU-DB). The intensity of each target protein band was divided by the corresponding intensity of the β-actin band internal control and statistically normalized data were used to comparing between groups. All experiments were performed in triplicates, and the results have been presented as mean ± standard deviation.

**Figure 12:**
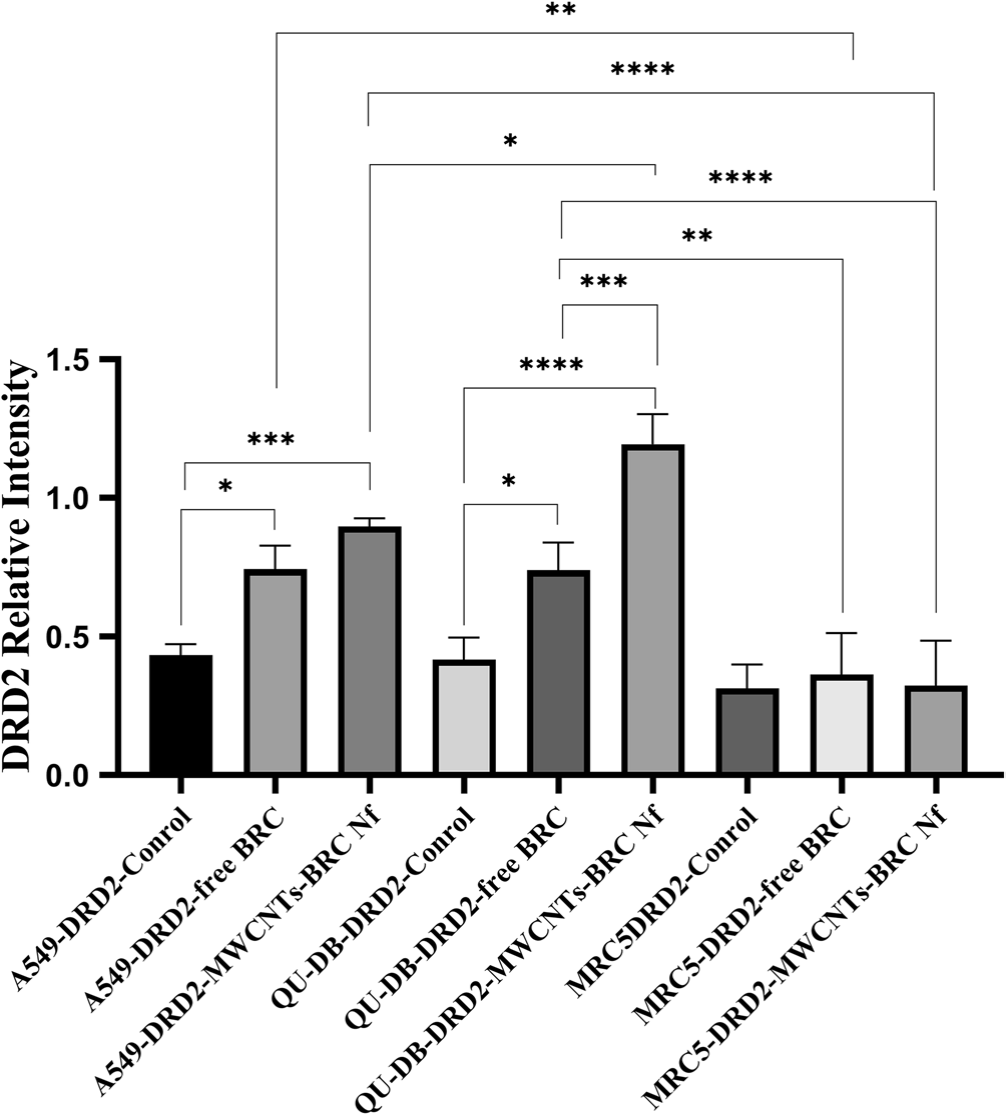
Comparison of DRD2 relative protein expression levels in lung cancer cell lines (A549 and QU-DB) and normal cell lines (MRC5). The intensity of each target protein band was divided by the corresponding intensity of the β-actin band internal control and statistically normalized data were used to comparing between groups. All experiments were performed in triplicates, and the results have been presented as mean ± standard deviation.

**Figure 13:**
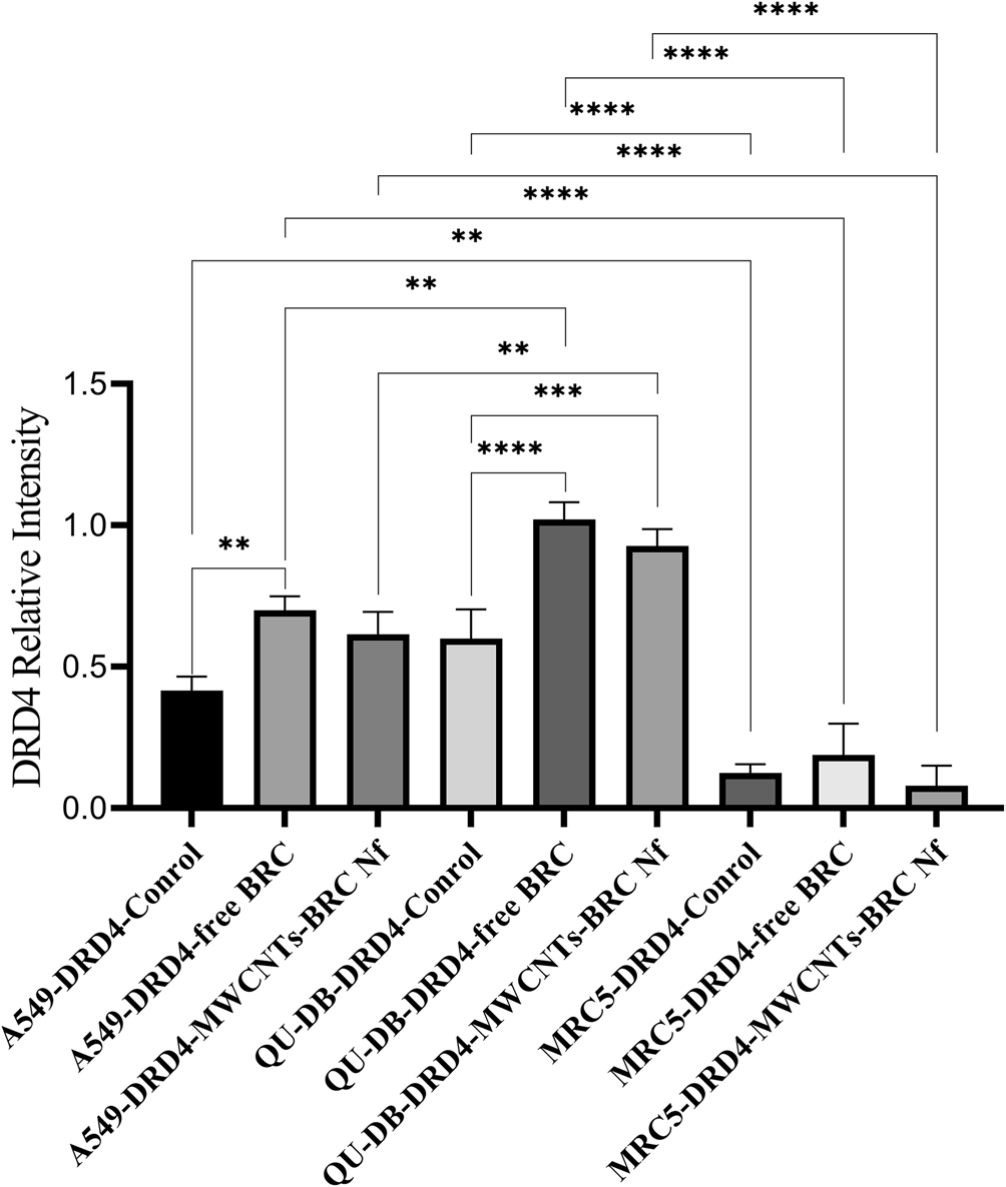
Comparison of DRD4 relative protein expression levels in lung cancer cell lines (A549 and QU-DB) and normal cell lines (MRC5). The intensity of each target protein band was divided by the corresponding intensity of the β-actin band internal control and statistically normalized data were used to comparing between groups. All experiments were performed in triplicates, and the results have been presented as mean ± standard deviation.

**Figure 14:**
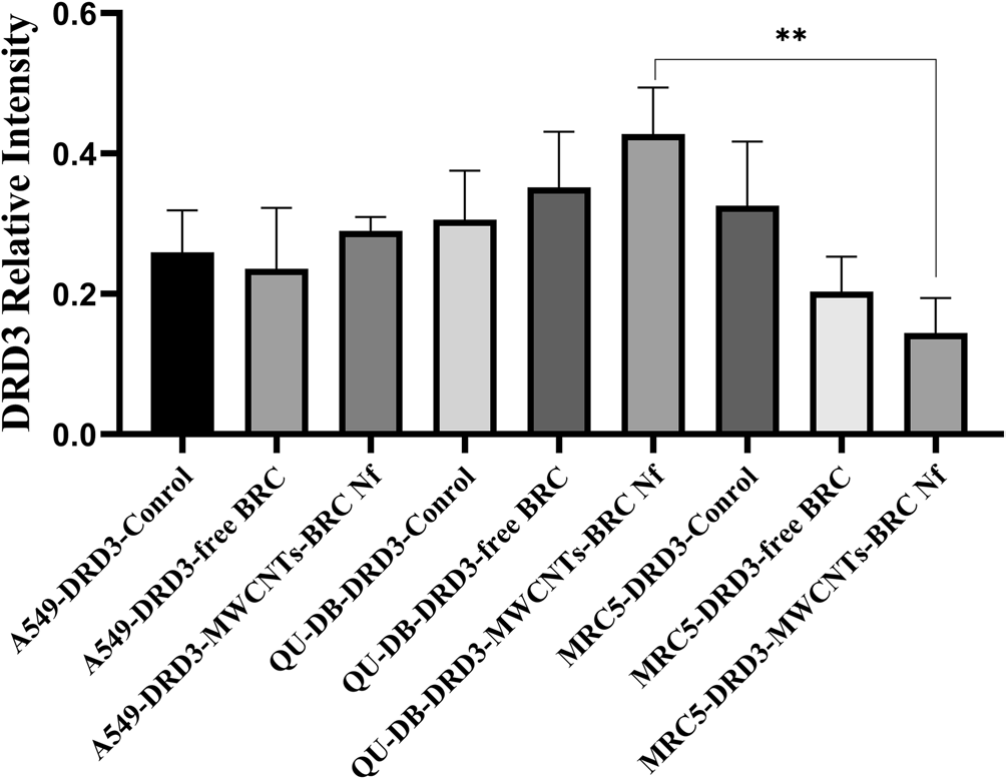
Comparison of DRD3 relative protein expression levels in lung cancer cell lines (A549 and QU-DB) and normal cell lines (MRC5). The intensity of each target protein band was divided by the corresponding intensity of the β-actin band internal control and statistically normalized data were used to comparing between groups. All experiments were performed in triplicates, and the results have been presented as mean ± standard deviation.

**Figure 15:**
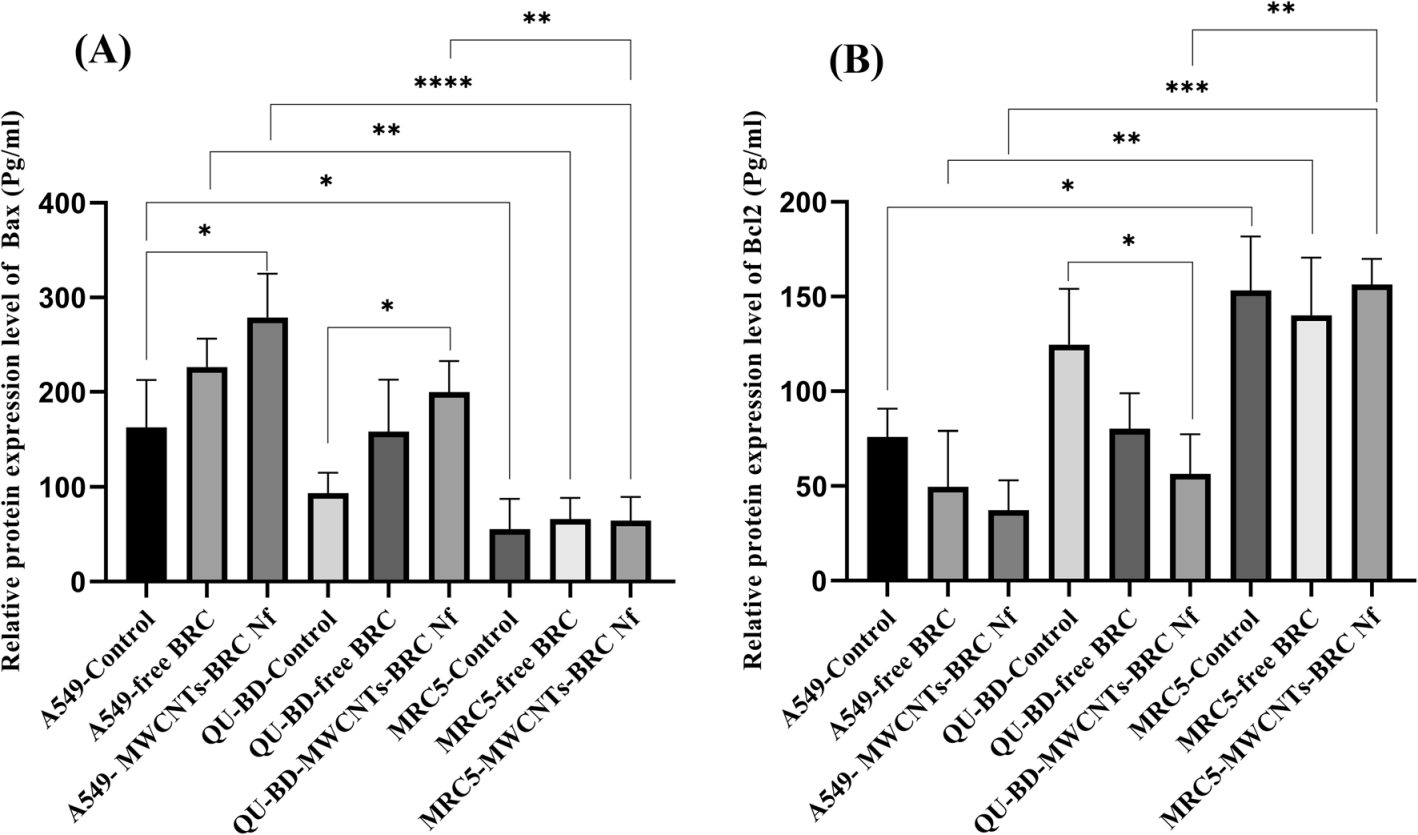
ELISA analysis of the relative expression level of Bax (**A**) and Bcl2 (**B**) associated proteins: data analysis demonstrated increased Bax protein levels in lung cancer cell lines (A549 and Qu-DB) vs lung normal cell line (MRC5), while on the contrary, decreased Bcl2 protein levels in lung cancer cell lines (A549 and Qu-DB) vs lung normal cell line (MRC5) cells was observed (P < 0.05).

Western blot analysis was performed to in-vitro assess the relative protein levels of dopamine receptors (D1–D5) in normal and lung cancer cells (Fig. 10) (S5–S10). As shown in Figs. 11, 12, 13 and 14, DRD2 and DRD4 receptors were significantly higher than other DRDs in treated (free BRC and MWCNTs-BRC Nf) lung cancer cell lines compared to untreated groups. According to one-way ANOVA, there were no significant differences between the intensity of each relative protein level of DRD2 receptors in untreated lung cancer cells, as well as treated or untreated MRC5 normal cell line. The expression levels of DRD2 receptors were significantly higher in nano drug-treated lung cancer cell lines compared to treated or untreated MRC5 normal cell lines. Also, the expression levels of DRD2 in BRC-treated lung cancer cell lines were significantly higher compared to treated or untreated MRC5 normal cell lines. Importantly, the expression levels of the DRD2 nano drug-treated QU-DB lung cancer cell line were significantly higher compared to the A549 lung cancer cell line. The expression levels of DRD4 (control, free BRC, and MWCNTs-BRC Nf) in cancer cell lines were higher significantly compared to MRC5 groups, and the expression levels of DRD4 were significantly higher in QU-DB lung cancer cell lines compared to A549 cell lines. The expression levels of DRD3 in the control groups of all cancer and normal cell lines were not considerably different and were almost the same. The expression levels of DRD3 in QU-DB cells, which were treated with nano-drug, were significantly higher compared to nano drug-treated MRC5 cell line. ELISA results of Bax (Bcl-2 associated X protein) and Bcl2 (B-cell lymphoma 2) protein expression levels in A549, QU-DB, and MRC5 cell lines after 48 h showed that in BRC and conjugated nano-drug treatments, the levels of Bax and Bcl2 in the three groups of cell lines were different. Bax was positively correlated with the level of apoptosis, whereas Bcl-2 was negatively correlated with the level of apoptosis (Fig. 15).

The Bax expression levels were higher in BRC- and nano drug-treated A549 and QU-DB cells compared to control cells, but they were only significant in nano drug-treated cells. There were no differences in Bax and Bcl-2 expression levels in the MRC5-treated cells compared to the control groups, and they showed almost the same levels, and overall, Bax and Bcl-2 expression levels were markedly different compared to A549 and QU-DB cells^24^.

### Transmission electron microscopy study

**Figure 16:**
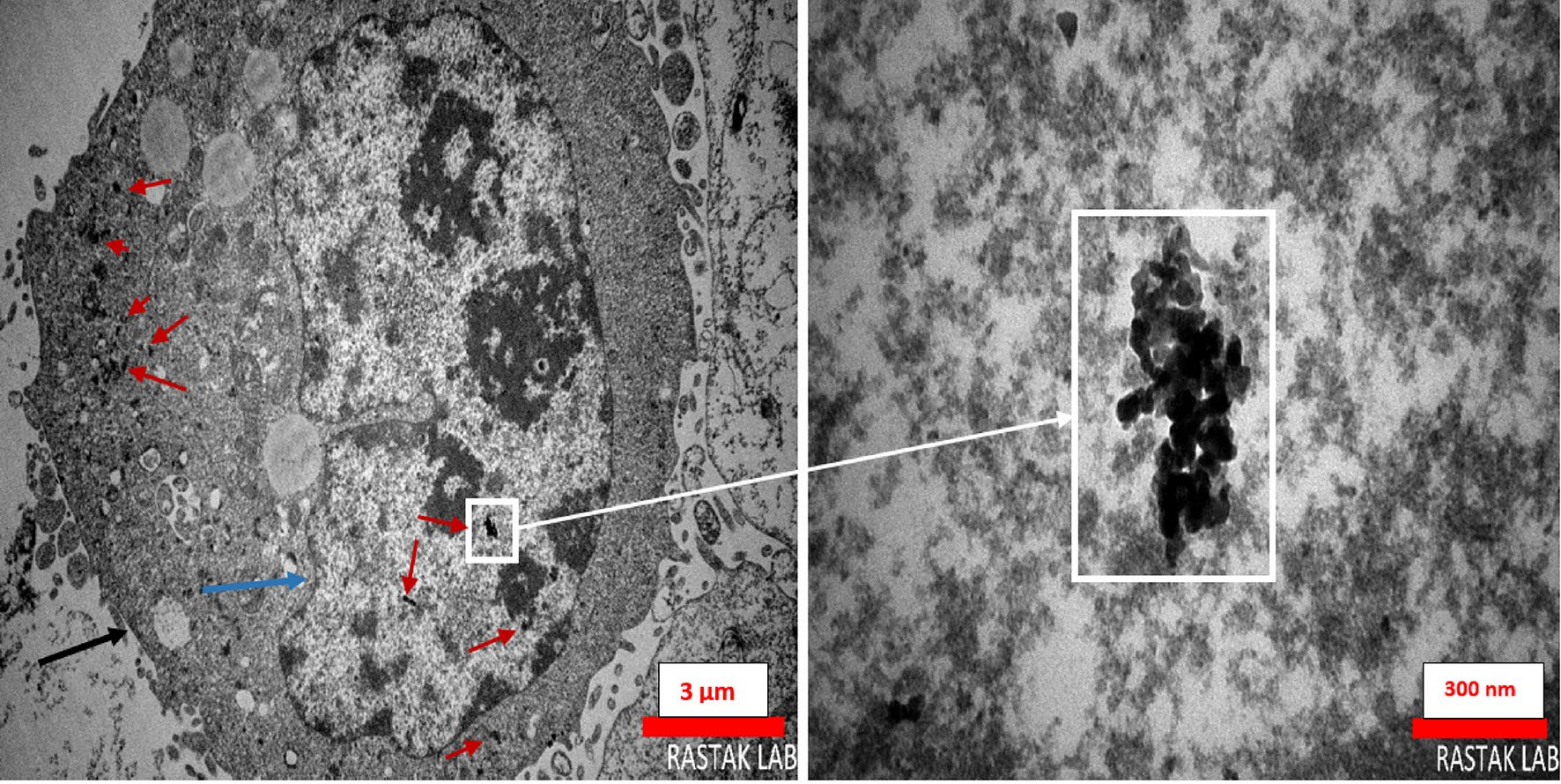
Characterization of MWCNTs-BRC Nf by TEM: cellular uptake and nuclear inserting of BRC-conjugated MWCNTs in A549 lung cancer cell line after 48 h treatment (50 μg/ml) and incubation at 37 °C. Arrows point to: white and red; MWCNTs, blue and black; cellular uptake processes. All scale bars represent in each image. Original, unprocessed images are presented in Supplementary Figures S11 and S12.

TEM results of MWCNTs-BRC Nf treatment are shown in Fig. 16 (S11, S12). The diameter of conjugated MWCNTs was about 25–40 nm. As sonication might cut nanotubes to shorter fragments, the average length of MWCNTs was about ∼ 500 nm. It has been reported that the uptake of carboxylated MWCNTs by HEK293, a non-phagocytic cell, occurs by two approaches, first direct penetration of single nanotubes via the plasma membrane, and second, through the uptake of MWCNTs cluster via endocytosis processes^25^. Due to their greater diameter, MWCNTs are capable of endosomal escape, piercing lysosomes, release into the cytoplasm, and interaction with the nucleus^26^. Short MWCNTs can penetrate the cell nucleus^25^.

## Discussion

In this study, a specific agonist of DRDs, BRC was delivered to lung cancer cells with MWCNTs and showed a significant anti-proliferative effect on cancer cells and low toxicity on normal lung cells^6,12,27,28^. Previous studies support the concept that the D2R agonist due to the inhibition of cancer progression can be used as a therapeutic agent. Besides, the developing number of studies point to the interaction between dopamine and the immune system. DRDs can act as modulators in the activities of both cellular and humoral immune systems^29^. The results of McKenna et al. showed relatively variable DRD2 and DRD4 on peripheral blood leukocytes expression, although DRD3 and DRD5 were expressed on the majority of leukocyte subsets. Moreover, DRs were expressed, at a constantly high level on B cells and NK cells, partially on neutrophils and eosinophils, and in a small level on T cells and monocytes^30^.

Recently, DRDs antagonists have been widely considered in cancer research, and the function of the dopamine receptor on bio-behavior of the tumor as a potential therapeutic target has been declared. Also, in different types of tumors, the more favorable therapeutic efficiency of agonists or antagonists of DRDs depends mainly on the high-level expression DRDs^1,31^. DRDs play a crucial role in cancer tumorigenesis and development. Camia et al. showed that the polymorphisms of DRD2 causing lower dopamine bioavailability are associated with a greater risk of non-small cell lung carcinoma (NSCLC)^27,32,33^. Campa et al. showed that the polymorphisms of DRD2 causing lower dopamine bioavailability are associated with a greater risk of non-small cell lung carcinoma (NSCLC)^34^.

A decrease in the expression levels of DRD2 in NSCLC tissues in comparison to adjacent normal lung tissues was negatively associated with the tumor size and total survival. DRD2 inhibits NSCLC cell growth by blocking the NF-κB signaling pathway both in vitro and in vivo^35^*.* Previous studies have shown that BRC as a specific agonist of DRDs exerted anticancer effects^6,12,13,36–38^. In this research, after treatment of lung cancer cells with BRC, high expression levels of DRD2 were found and at a low dosage of the drug in conjugated form, this effect was found to be stronger while having more potent anti-cancer effects and less toxicity.

The MTT results after 24 h of treatment with free BRC, MWCNT NFs, and targeted MWCNTs-BRC Nf showed more sensitivity to apoptosis in A549 and QU-DB cells compared to normal MRC5 cell line. Furthermore, the targeted NPs significantly induce more apoptosis and less viability compared to free BRC and MWCNTs. The results of the MTT assay after 48 h showed a more potent cytotoxicity effect of conjugated NPs than 24 h in both cancer cell lines compared to free BRC and F-MWCNTs Nf. A decrease in the IC_50_ value of targeted MWCNTs-BRC Nf formulation was estimated in lung cancer cells compared to the MRC5 cell line after 24 and 48 h. According to the results, MWCNTs-BRC Nf had a significant effect on decreasing the viability rate and IC_50_ values of cancer cell lines compared to the normal MRC5 cell line. This is due to the notable consequence of the DRD2s targeting in internalization of BRC into the cancer cell lines in addition to several parameters, such as high vehicle capacity, increase in the solubility and stability of functionalized MWCNTs, and needle-like structure of CNTs to cross the cell membrane^39,40^.

Accordingly, it can be concluded that the normal MRC5 cell line was significantly more resistant to apoptosis than A549 and QU-DB lung cancer cell lines and showed a higher viability rate against exposure to three different drugs. ELISA results of the relative expression levels of Bax and Bcl2 proteins confirmed our observations. Data analysis demonstrated increased Bax protein levels in lung cancer cell lines compared to MRC5, whereas decreased Bcl2 protein levels in lung cancer cell lines compared to MRC5 cells were observed. This could be due to the low expression of DRD2s on the MRC5 cell surface, which causes less interaction and drug uptake into the cell. Therefore, BRC as an agonist of DRDs conjugated by MWCNT NPs could have a synergic effect in lung cancer cells, making DRDs sensitive to programmed death, preventing their entry into the cell cycle, finally improving the cytotoxic effects.

Furthermore, it has been reported that cancer cells cause greater internalization of nanoparticles compared to normal cells because of the enhanced permeation and retention (EPR) effect^41^; therefore, because of the vascular permeability of tumors, nanoparticles are accumulated in the tumor cells compared to normal cells^42,43^. Several other studies have reported no damage or toxicity to healthy cells by CNTs^44,45^. DRD1–D5 mRNA and protein levels were detected by qRT-PCR and western blot analysis, respectively in all three cell lines.

We found that DRD2 expression levels in lung cancer cell lines and normal MRC5 were nearly the same before treatment; nevertheless, after cell treatment with drugs, the expression levels of this receptor altered and when it was exposed to BRC, the expression levels of this receptor in cancer cells compared to normal MRC5 increased significantly and after treatment with targeted nano drag, particularly in QU-DB cells, a further increase was observed. Regarding DRD4, the expression levels were significantly higher in cancer cells than in normal cell lines. After treatment with BRC and targeted nano-drug, increased expression levels were observed in both cancer cell lines; however, it was significant in QU-DB cells. Concerning the expression levels of receptor 3 before and after treatment, no significant difference was observed in cancer cells compared to normal cells, except in QU-DB cells that were exposed to targeted nano drag. Also, the expression levels of DRD1 and DRD5 genes did not show any significant changes. Our observations are in agreement with previous studies about the expression levels of DRDs and the role of their agonist in lung cancer^27,35,46^. Accordingly, it is critical to identify the exact pharmacological and toxicological properties of CNTs before utilizing them in medical settings. Surface functionalization and modification of CNTs are appropriate methods to reduce their cytotoxicity, overcome limitations, such as distribution, accumulation rate, and clear pathways for CNT-based drug delivery systems. Hence, approaches for surface functionalization comprising covalent and noncovalent functionalization are employed to synthesize CNTs^47^.

Functionalization is also helpful in conjugating the therapeutic molecule or the ligands on the surface or ends of CNTs to make them active against cancer cells^40^. In this study, we demonstrated that CNT-based drug delivery possesses a magnificent potential for cancer therapy. Two basic properties have made carbon nanotubes suitable agents, especially for use and clinical purposes in drug delivery. One of these features is the ability to penetrate cell walls and increase drug load without causing cell death. Another feature is their biocompatibility so that after drug delivery, nanotubes can be eliminated from the systemic circulation through the renal excretory pathway without side effects^48–50^.

Herein, we reported the first active targeted, ligand-receptor BRC-conjugated MWCNTs and demonstrated in vitro killing of cancer cells, enhanced anticancer effects, and reduced cytotoxicity of the drug at a high dose in the normal cell line. Targeted drug delivery systems can improve the protection of healthy cells from the cytotoxic compounds, minimize drug threshold dose and inhibit adverse effects, and reduce the drug-resistant cancerous cells^51^.

In conclusion, in our previous study, it was found that the combination treatment with BRC and MWCNTs nano-fluid (MWCNTs Nf) could inhibit the proliferation of A549 and QU-DB cells through the induction of apoptosis in addition to necrotic effects. The present study was designed to further investigate the mechanism of suppression of lung cancer cell growth, as well as eliminate the necrotic effects, which seemed to be due to the synergistic and excessive effects of these compounds. To our knowledge, for the first time, we designed an active targeted BRC-conjugated functionalized MWCNTs nanofluid drug delivery system to investigate its efficiency on lung cancer cell lines. In this article, the functionalized MWCNTs Nf as a drug delivery system could improve the efficiency of the anti-tumor activity as well as reduce drug dosage and eventually decrease the side effects of BRC.

## Supplementary Information

Supplementary Information.
