# Supplementary material for: A success targeted nano delivery to lung cancer cells with multi-walled carbon nanotubes conjugated to bromocriptine
Source: Sci Rep. 2021 Dec 24;11:24419. doi: 10.1038/s41598-021-03031-2 (PMC8709863; doi:10.1038/s41598-021-03031-2)
Supplement: Supplementary file 1 — Supplementary Information. [file 41598_2021_3031_MOESM1_ESM.docx]

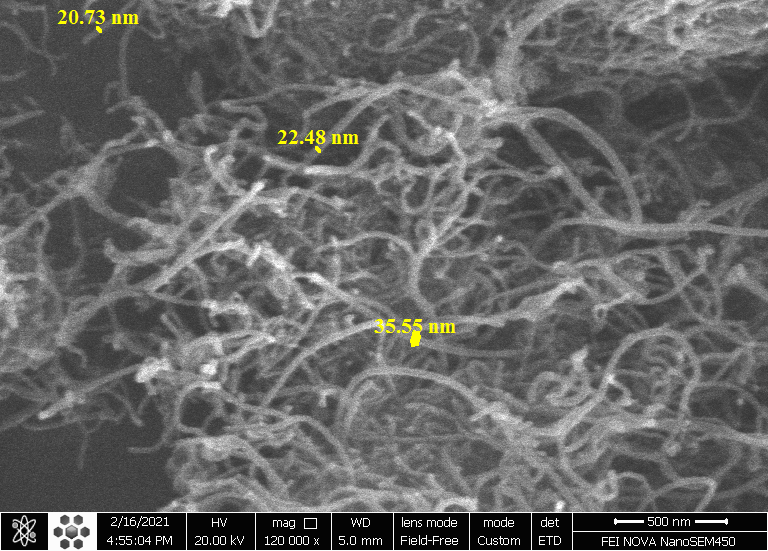


**S1:** SEM image of the functionalized MWCNTs (500nm)


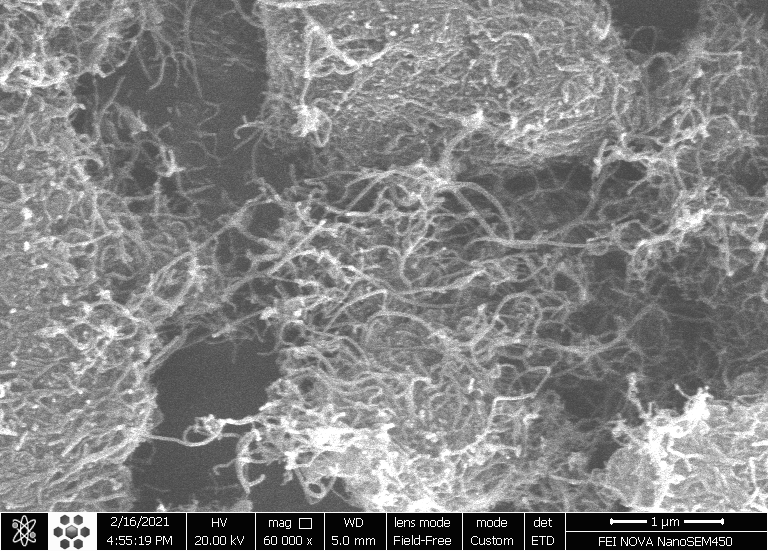


**S2:** SEM image of the functionalized MWCNTs (1µm)


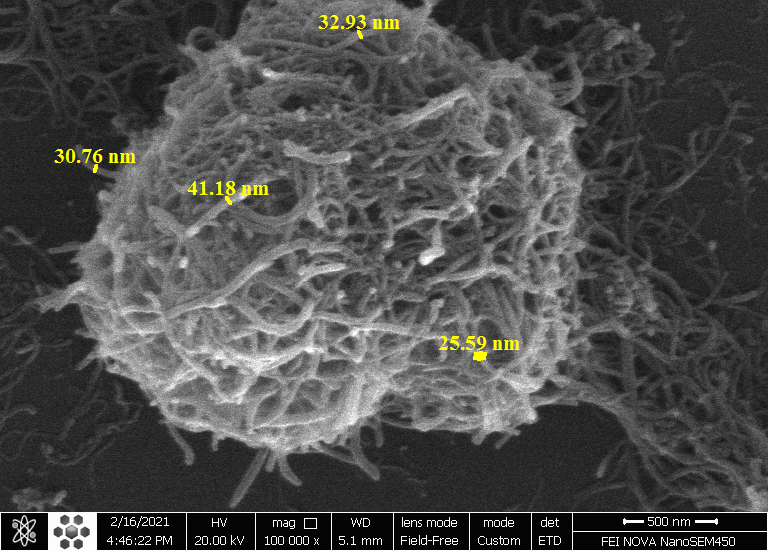


**S3:** SEM image of conjugated MWCNTs – BRC (500nm)


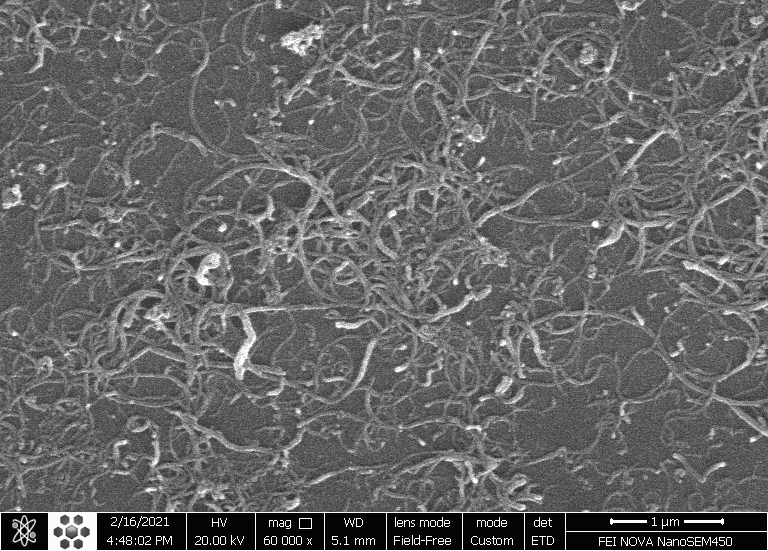


**S4:** SEM image of conjugated MWCNTs – BRC (1µm)


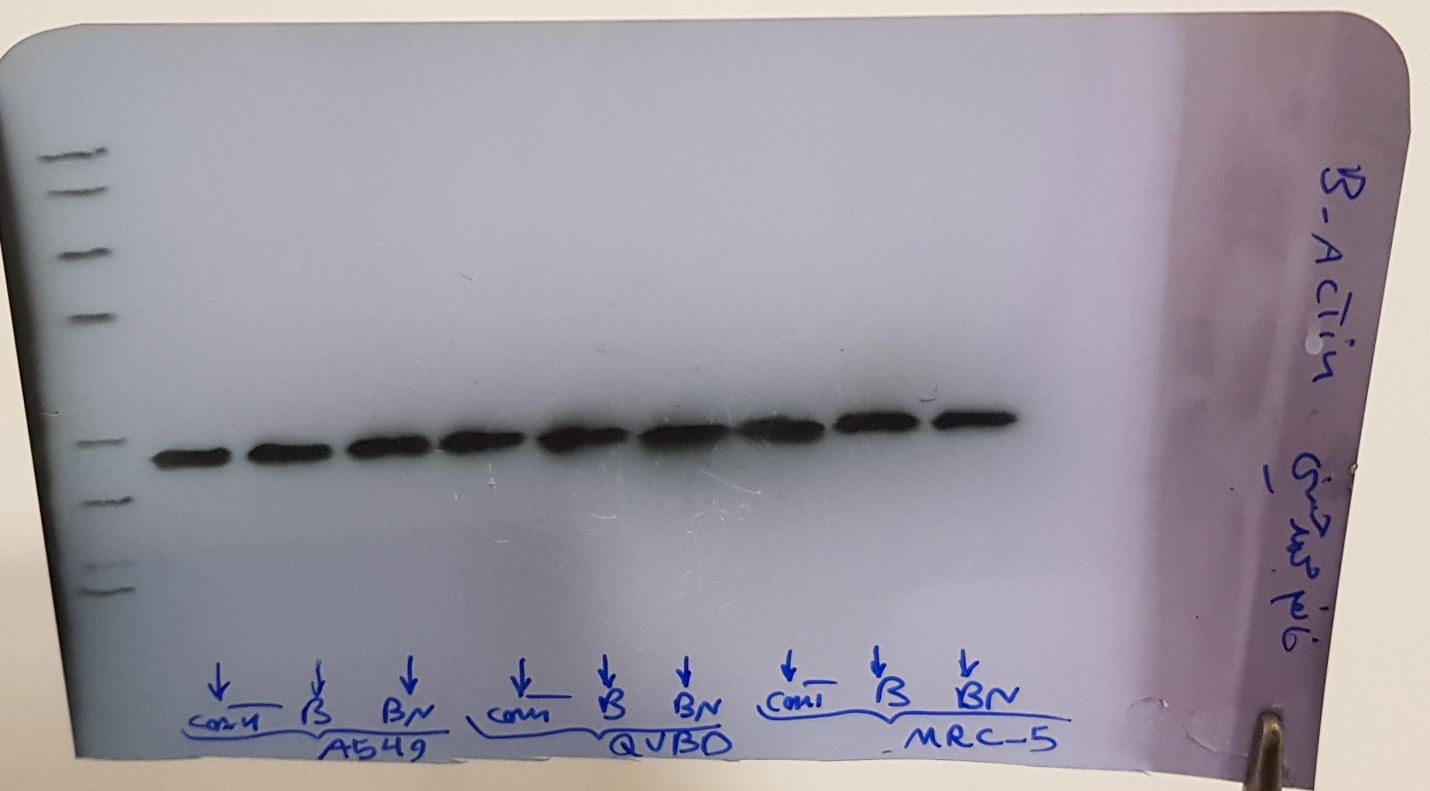


**S5:** Comparison of β-actin relative protein expression levels in lung cancer cell lines (A549 and QU-DB) and normal cell lines (MRC5). From left to right for each cell line: Control or untreated cells, treated cells with BRC and treated cells with MWCNTs-BRC.


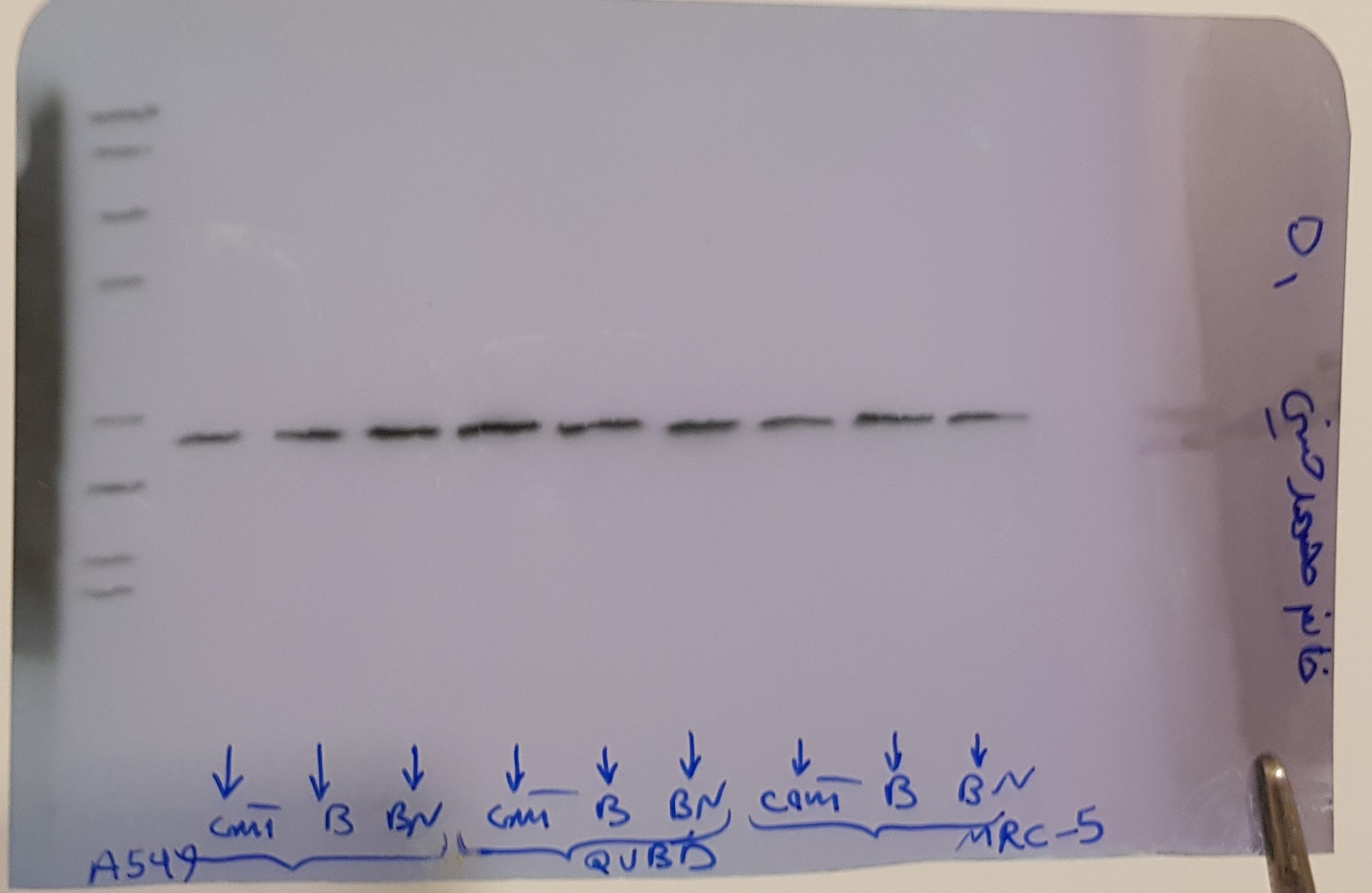


**S6:** Comparison of DRD1 relative protein expression levels in lung cancer cell lines (A549 and QU-DB) and normal cell lines (MRC5). From left to right for each cell line: Control or untreated cells, treated cells with BRC and treated cells with MWCNTs-BRC.


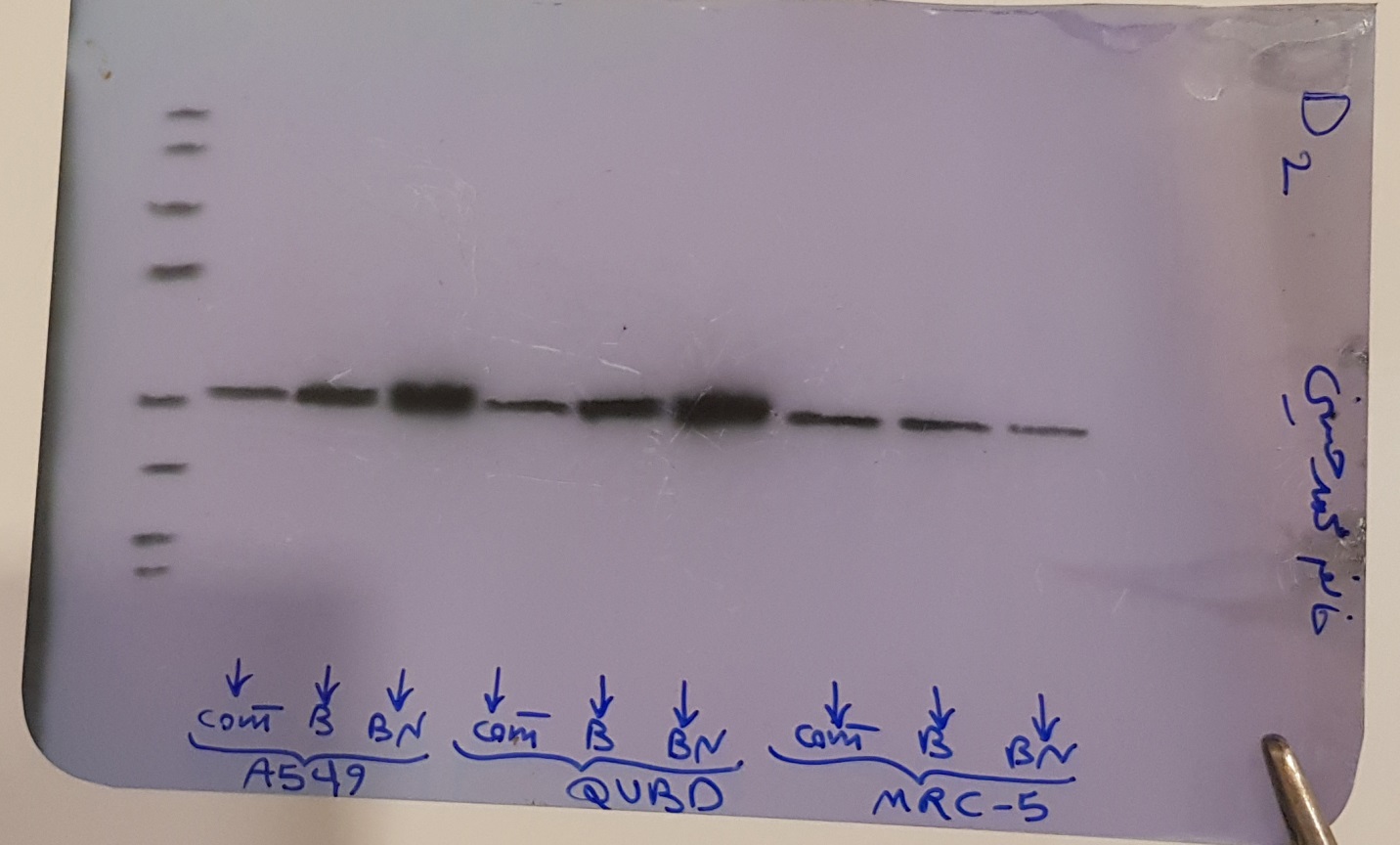


**S7:** Comparison of DRD2 relative protein expression levels in lung cancer cell lines (A549 and QU-DB) and normal cell lines (MRC5). From left to right for each cell line: Control or untreated cells, treated cells with BRC and treated cells with MWCNTs-BRC.


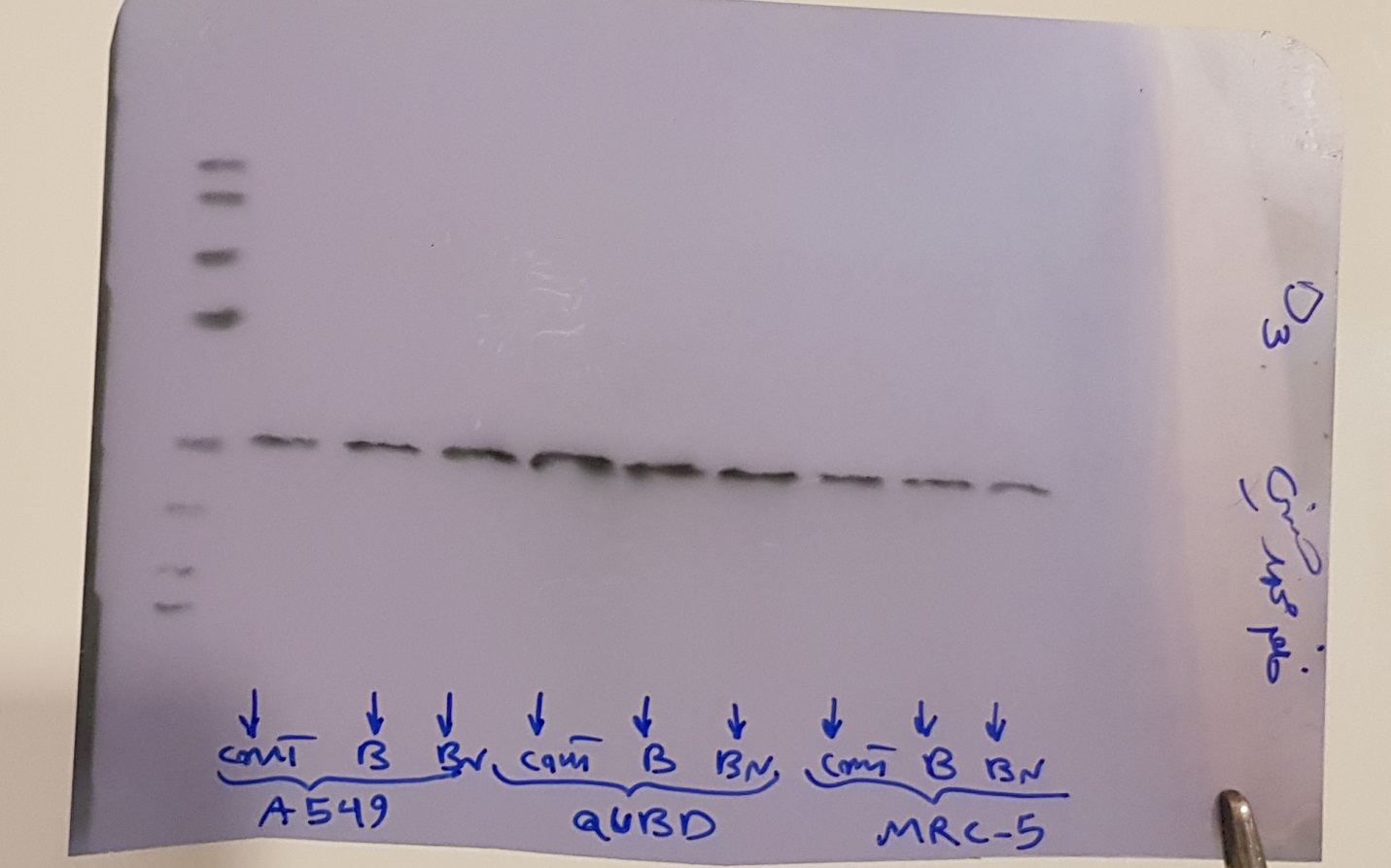


**S8:** Comparison of DRD3 relative protein expression levels in lung cancer cell lines (A549 and QU-DB) and normal cell lines (MRC5). From left to right for each cell line: Control or untreated cells, treated cells with BRC and treated cells with MWCNTs-BRC.


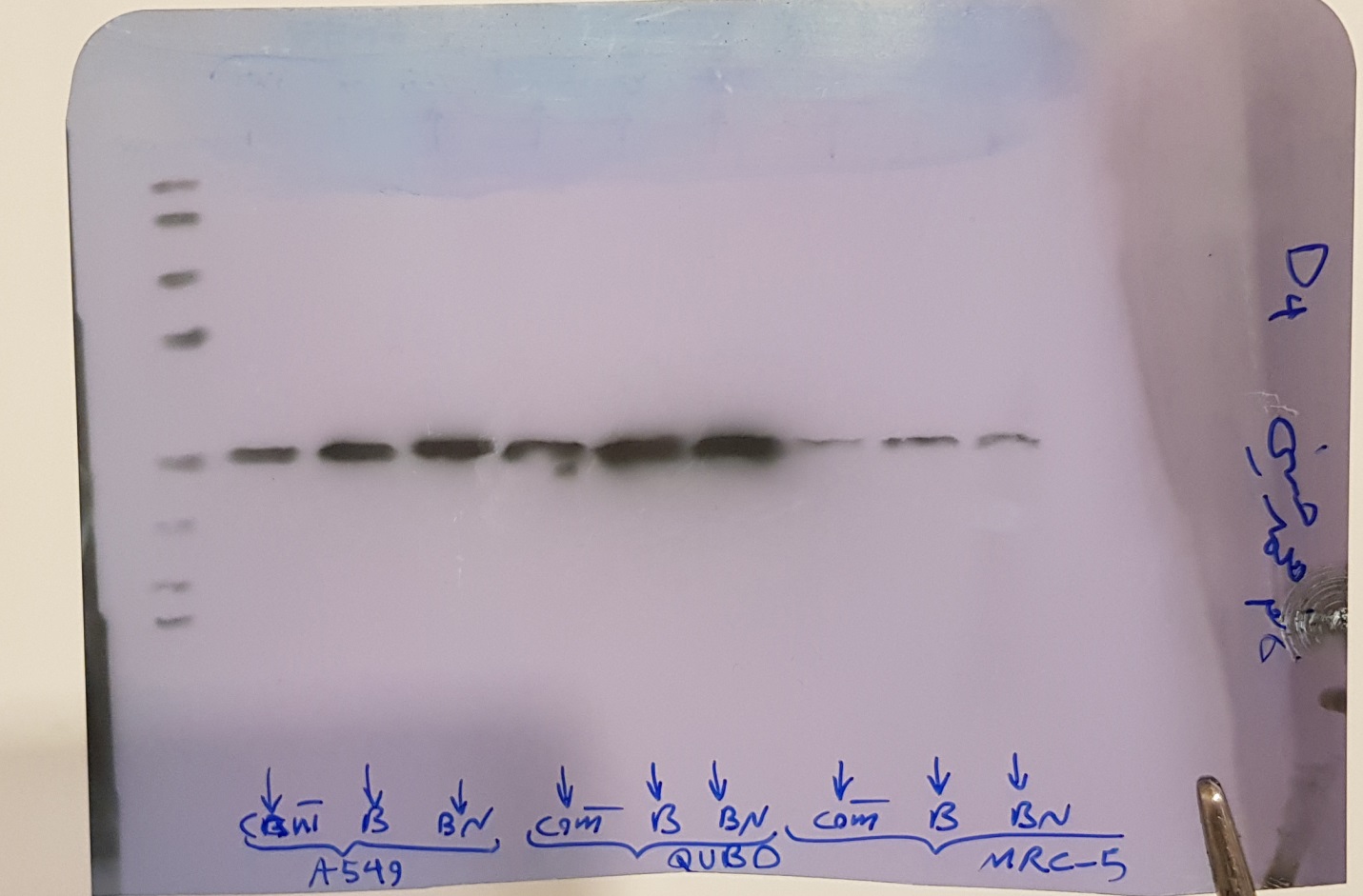


**S9:** Comparison of DRD4 relative protein expression levels in lung cancer cell lines (A549 and QU-DB) and normal cell lines (MRC5). From left to right for each cell line: Control or untreated cells, treated cells with BRC and treated cells with MWCNTs-BRC.


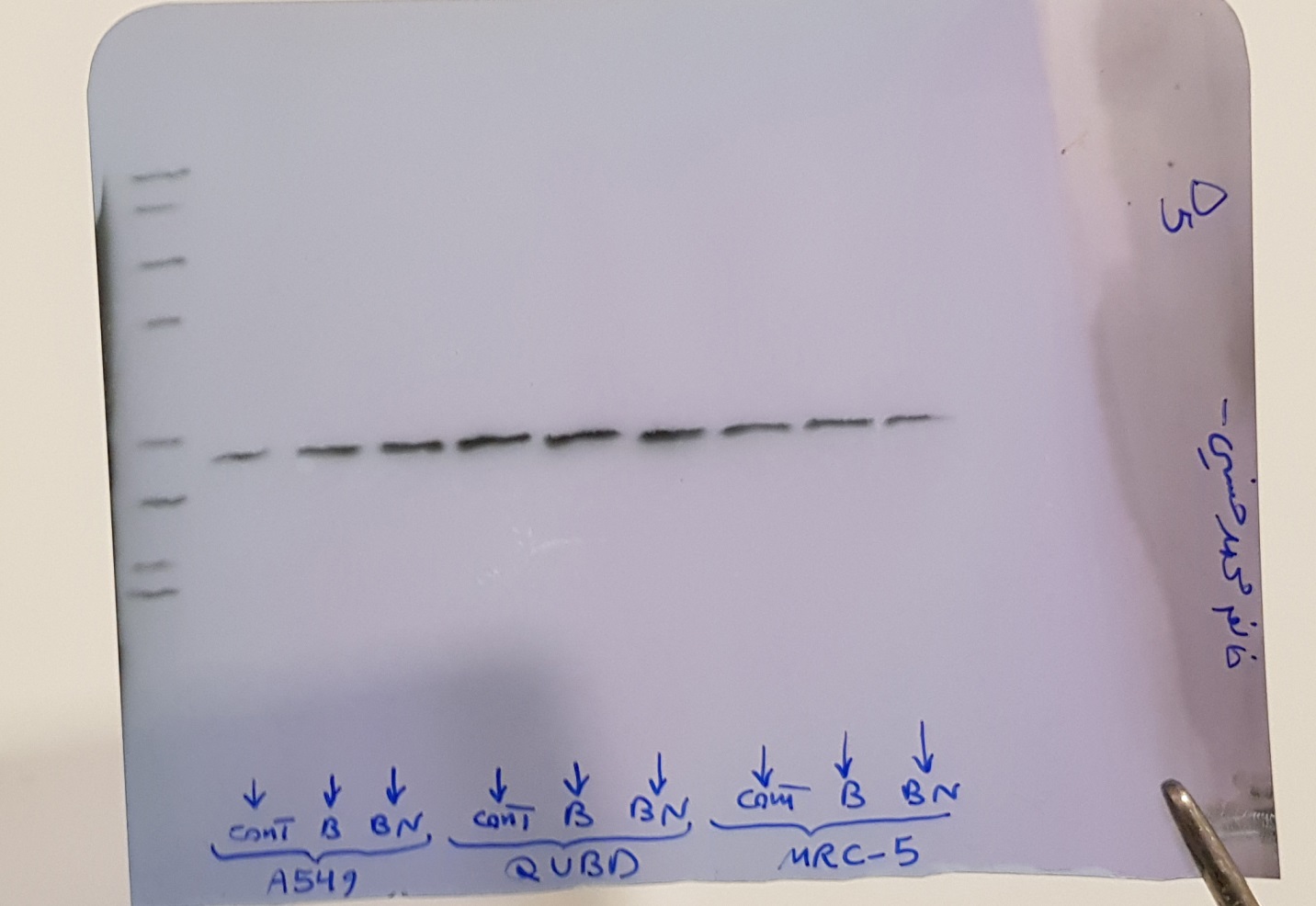


**S10:** Comparison of DRD5 relative protein expression levels in lung cancer cell lines (A549 and QU-DB) and normal cell lines (MRC5). From left to right for each cell line: Control or untreated cells, treated cells with BRC and treated cells with MWCNTs-BRC.


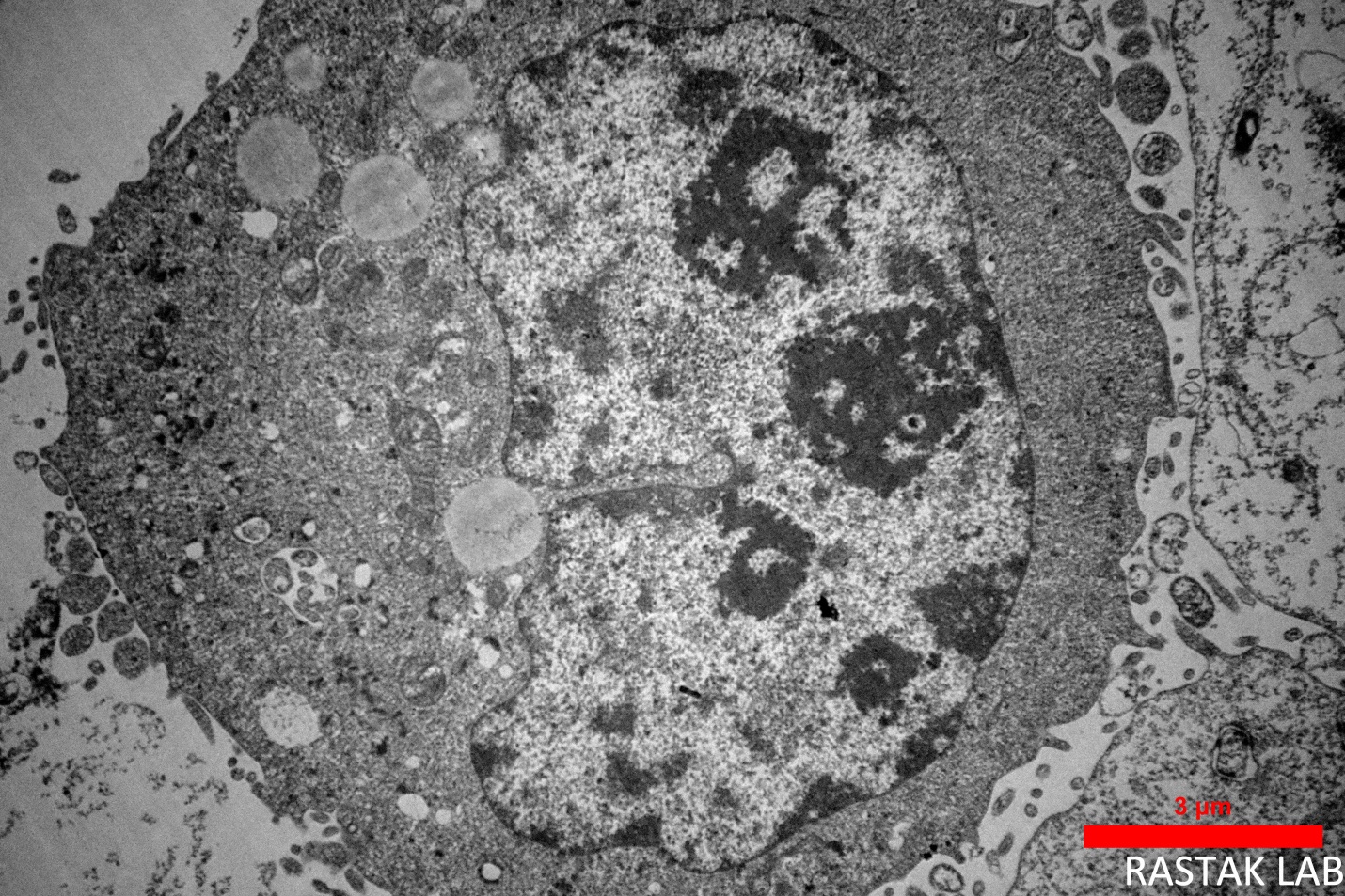


**S11:** Characterization of MWCNTs-BRC Nf by TEM, Cellular uptake and nuclear inserting of BRC-conjugated MWCNTs in A549 lung cancer cell line after 48h treatment (50 μg/ml) and incubation at 37 ^o^C(3µm).


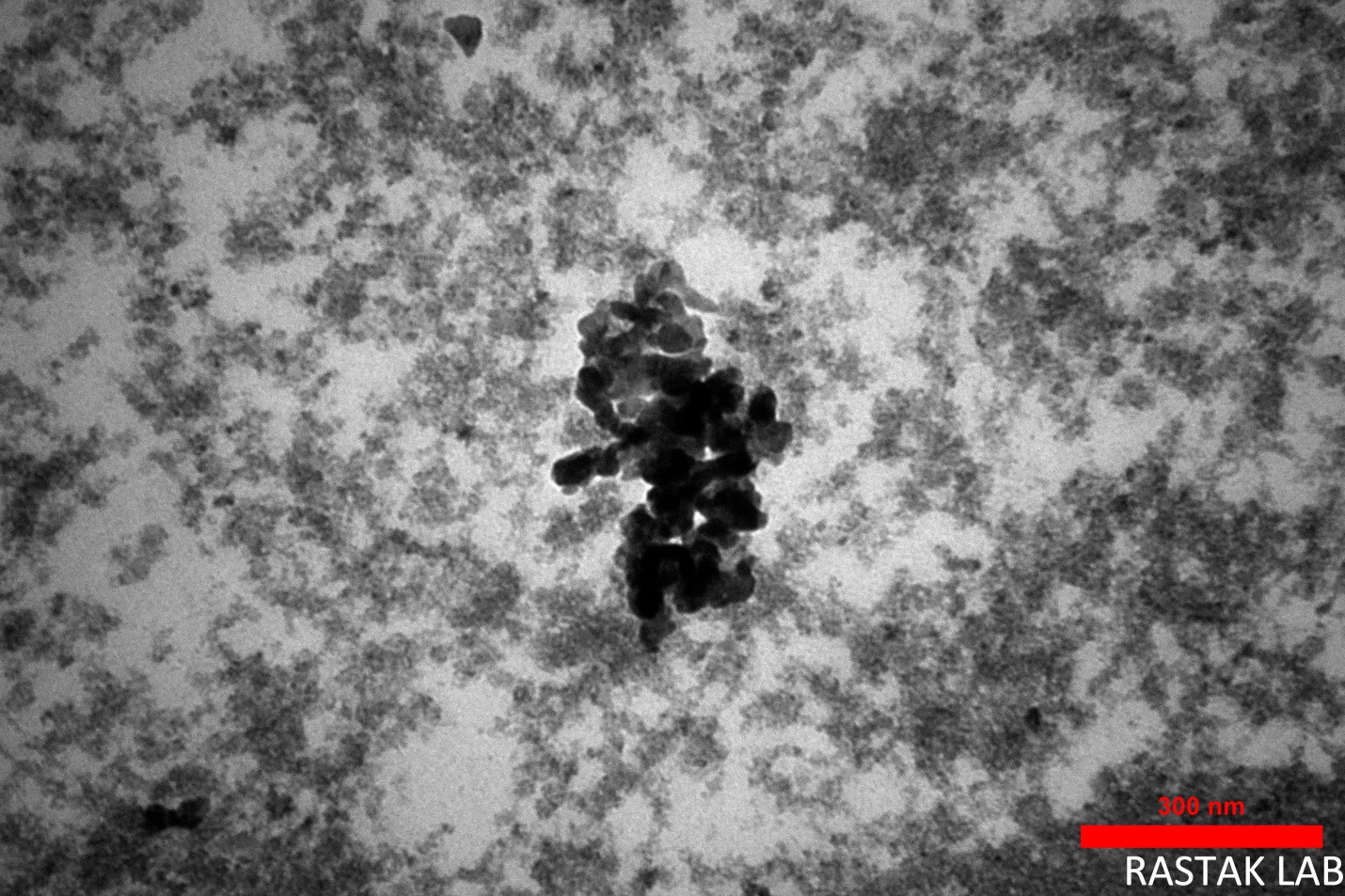


**S12:** Characterization of MWCNTs-BRC Nf by TEM, Cellular uptake and nuclear inserting of BRC-conjugated MWCNTs in A549 lung cancer cell line after 48h treatment (50 μg/ml) and incubation at 37 ^o^C, (300nm)
